# Empagliflozin Modulates Apoptosis and Autophagy Pathways in Lung Cancer Through Network Pharmacology and In Vitro Analysis

**DOI:** 10.1002/jbt.71174

**Published:** 2026-11-07

**Authors:** Ola A. El‐Feky, Sara M. Hamouda, Manar Mohammed El Tabaa, Sally E. Abu‐Risha, Ahmed B. M. Mehany, Othman F. Abdelzaher, Samar A. El‐Adawy

**Affiliations:** ^1^ Biochemistry Department, Faculty of Pharmacy Tanta University Tanta Egypt; ^2^ Department of Biochemistry, Faculty of Pharmacy Alsalam University Kafr El Egypt; ^3^ Pharmacology & Environmental Toxicology, Environmental Studies & Research Institute (ESRI) University of Sadat City Sadat City Egypt; ^4^ Pharmacology Department, Faculty of Pharmacy Tanta University Tanta Egypt; ^5^ Zoology Department, Faculty of Science Al‐Azhar University Cairo Egypt

**Keywords:** apoptosis, autophagy, empagliflozin, hydroxychloroquine, LC3‐II, lung cancer, p62, SGLT2 inhibitor

## Abstract

Lung cancer remains a major cause of cancer‐related mortality, highlighting the need for novel therapeutic strategies. Empagliflozin (EMPA), a sodium–glucose cotransporter 2 inhibitor, has demonstrated emerging anticancer activity; however, its effects on apoptosis–autophagy crosstalk in lung cancer remain incompletely understood. This study investigated the anticancer effects of EMPA and the influence of hydroxychloroquine (HCQ)‐mediated autophagy inhibition in A549 and HOP‐92 human lung cancer cells. Network pharmacology with explicit database association‐score cutoffs, and molecular docking validated by redocking of a co‐crystallized reference ligand, were used to identify potential EMPA‐associated targets and pathways. Cytotoxicity of EMPA, HCQ, and their combination was evaluated by MTT assay, followed by combination index and Bliss analyses. Cell‐cycle distribution and apoptosis were assessed by flow cytometry. Expression of apoptosis‐ and autophagy‐related markers was evaluated by RT‐qPCR, cleaved caspase‐3 analysis, and Western blotting. Network analysis identified 239 overlapping EMPA–lung cancer targets, of which 46 were retained after applying an Open Targets association‐score cutoff of 0.30. Docking, validated by reproducing the erlotinib crystal pose in the EGFR kinase domain (heavy‐atom RMSD 1.30 ± 0.02 Å), indicated that EMPA can be accommodated at surface sites of ANXA8 (−7.6 kcal/mol) and the p62/SQSTM1 ZZ domain (−7.0 kcal/mol) and within the ligand‐binding pocket of PPARγ (−7.5 kcal/mol; PDB 8BF1, the PPARγ–PGC‐1α coactivator complex). EMPA reduced cell viability in both cell lines, with greater sensitivity in A549 cells. Combining EMPA with HCQ markedly reduced IC_50_ values and demonstrated synergistic interactions at IC_50_. EMPA altered cell‐cycle progression and increased apoptotic cell death, accompanied by enhanced BAX and CASP3 expression and cleaved caspase‐3 activation. EMPA increased LC3‐II without p62 accumulation, consistent with increased autophagic activity, whereas HCQ‐containing treatments induced LC3‐II and p62 accumulation and enhanced apoptotic signaling. EMPA exerts antiproliferative and pro‐apoptotic effects in lung cancer cells while modulating autophagy. Inhibition of lysosomal autophagic degradation by HCQ enhances EMPA‐mediated cytotoxicity and apoptosis, supporting further investigation of this combination as an anticancer strategy.

## Graphical abstract

An integrated computational and *in vitro* approach was used to investigate the anticancer activity of empagliflozin (EMPA) in A549 and HOP‐92 lung cancer cells and its interaction with hydroxychloroquine (HCQ). Network pharmacology identified 239 shared EMPA–lung cancer targets, while molecular docking predicted favorable interactions with ANXA8, SQSTM1/p62, and PPARGC1A. Experimentally, EMPA reduced cell viability, altered cell‐cycle progression, and promoted apoptosis, as evidenced by increased BAX, CASP3, and cleaved caspase‐3. EMPA also increased LC3‐II without marked p62 accumulation, consistent with an autophagy‐associated response. HCQ‐mediated inhibition of lysosomal autophagic degradation enhanced EMPA cytotoxicity and apoptotic signaling, supporting functional crosstalk between autophagy and apoptosis in the response of lung cancer cells to EMPA.

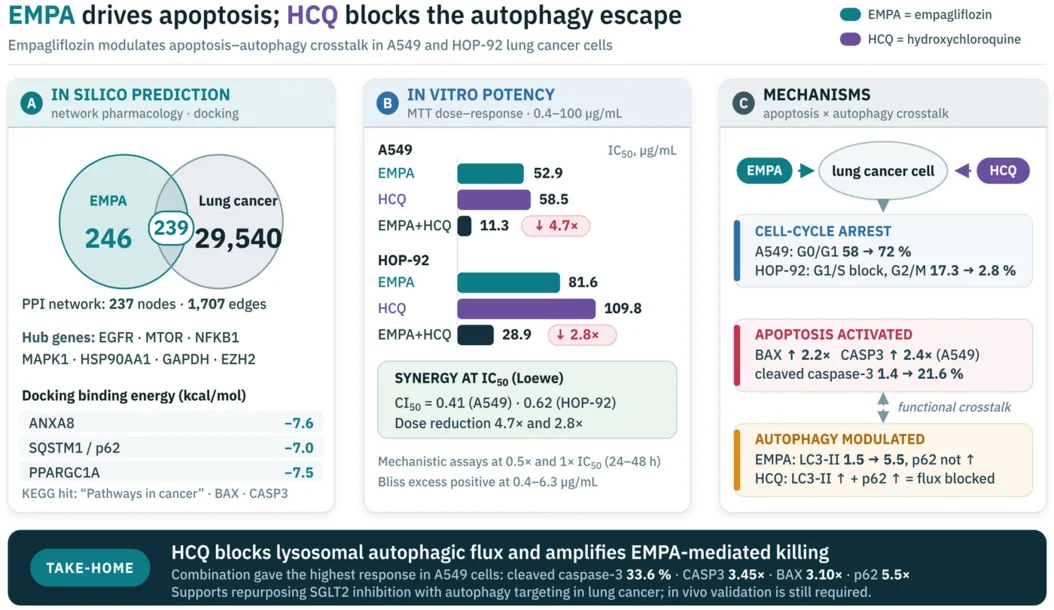

## 1 Introduction

Lung cancer remains one of the most common malignancies and a leading cause of cancer‐related mortality worldwide. Histologically, it is broadly classified into small‐cell lung cancer (SCLC) and non‐small‐cell lung cancer (NSCLC), with NSCLC accounting for approximately 85% of cases. NSCLC comprises several major histological subtypes, including adenocarcinoma, squamous cell carcinoma, and large‐cell carcinoma, with adenocarcinoma representing the most prevalent subtype [1, 2]. Despite substantial advances in molecularly targeted therapies and immunotherapy, therapeutic resistance and disease progression continue to limit long‐term clinical outcomes in NSCLC [1, 2]. This highlights the need to identify additional therapeutic strategies capable of targeting the molecular mechanisms that support lung cancer cell survival.

Among these mechanisms, apoptosis and autophagy play particularly important and interconnected roles in determining cancer‐cell fate. Apoptosis is a tightly regulated form of programmed cell death that eliminates damaged or stressed cells, whereas autophagy is a lysosome‐dependent degradative process that maintains cellular homeostasis through the recycling of damaged proteins and organelles. In established tumors, autophagy may enable cancer cells to adapt to metabolic stress, nutrient deprivation, hypoxia, and anticancer treatment; however, excessive or context‐dependent autophagy may also contribute to cell death [3] (Levy et al. 2017). Accordingly, the functional relationship between autophagy and apoptosis is complex, and modulation of one pathway can substantially influence the other.

This interplay is particularly relevant to NSCLC, in which autophagy has been implicated in tumor progression and therapeutic resistance. Treatment‐induced autophagy may function as a cytoprotective mechanism that allows cancer cells to survive cellular stress, whereas inhibition of autophagic degradation can increase susceptibility to apoptotic cell death [3, 4]. Consequently, pharmacological targeting of autophagy has emerged as a potential strategy for increasing the sensitivity of cancer cells to anticancer treatment. Hydroxychloroquine (HCQ), a lysosomotropic agent, interferes with lysosomal function and thereby inhibits late‐stage autophagic degradation. Its use as an experimental autophagy inhibitor has therefore provided an important approach for investigating whether treatment‐induced autophagy contributes to cancer‐cell survival [5].

Empagliflozin (EMPA) is a selective sodium–glucose cotransporter 2 (SGLT2) inhibitor clinically established for the management of type 2 diabetes and associated cardiorenal disorders. Increasing experimental evidence suggests that the biological effects of SGLT2 inhibitors extend beyond glucose lowering and may include modulation of cancer‐cell metabolism, proliferation, survival, and stress‐response signaling. SGLT2 and related glucose‐transport mechanisms have therefore attracted interest as potential metabolic vulnerabilities in malignant cells (Wright et al. 2022).

Several preclinical studies have reported anticancer effects of EMPA and other SGLT2 inhibitors in different tumor models. SGLT2 inhibition has been associated with reduced proliferation and increased apoptosis in cervical cancer cells through AMPK‐associated signaling [6]. EMPA has also demonstrated antitumor activity in hepatocellular carcinoma, particularly when combined with metformin [7]. More recently, SGLT2 inhibitors have been shown to influence the chemosensitivity of breast cancer cells, further supporting their potential application as components of combination anticancer strategies [8]. These findings suggest that SGLT2 inhibition may affect cancer cells through mechanisms extending beyond systemic glucose control.

The potential involvement of AMPK/mTOR signaling is particularly relevant because this pathway links cellular energy status with proliferation, apoptosis, and autophagy. Experimental evidence indicates that EMPA can influence AMPK/mTOR‐associated signaling in cancer cells (Nasiri et al. 2022). Since mTOR is a major negative regulator of autophagy, alterations in this pathway may provide a mechanistic connection between the metabolic effects of EMPA and changes in autophagic activity. Nevertheless, the precise relationship among EMPA‐induced metabolic stress, autophagy, and apoptotic cell death remains incompletely defined, particularly in lung cancer.

The role of autophagy during EMPA treatment is especially important because increased autophagic activity may have different biological consequences depending on the cellular context. In cancer cells, it may represent an adaptive response that maintains energy homeostasis and limits treatment‐induced damage, potentially reducing apoptotic susceptibility [3, 4]. Conversely, disruption of this adaptive process may increase cellular stress and facilitate apoptotic execution. Thus, investigating EMPA in the presence of an autophagy inhibitor such as HCQ may help clarify whether autophagic/lysosomal activity influences the response of lung cancer cells to EMPA.

In this context, network pharmacology and molecular docking provide complementary approaches for identifying potential molecular targets and signaling networks through which EMPA may exert anticancer activity. Network‐based analysis is particularly suitable for drug repurposing because it can reveal interactions between a pharmacological agent and multiple disease‐associated targets rather than assuming a single‐target mechanism. Integration of these computational predictions with experimental validation can consequently provide a broader mechanistic understanding of drug action.

Therefore, the present study investigated the anticancer effects of EMPA in A549 and HOP‐92 human lung cancer cells using an integrated computational and experimental approach. Network pharmacology and molecular docking were employed to identify potential EMPA‐associated targets and pathways relevant to lung cancer, followed by in vitro assessment of cell viability, cell‐cycle distribution, apoptosis, apoptotic signaling, and autophagy‐associated markers. Furthermore, the effect of combining EMPA with HCQ was evaluated to determine whether interference with lysosomal autophagic degradation modifies EMPA‐induced cytotoxicity and apoptosis. This integrated approach was designed to clarify the interplay between EMPA‐induced apoptosis and autophagy and to provide a mechanistic basis for further investigation of EMPA‐based combination strategies in lung cancer.

## 2 Materials and Methods

### 2.1 Network Analysis

#### 2.1.1 Obtaining the Molecular Targets Associated With Empagliflozin

The chemical structure of empagliflozin, in Canonical SMILES format, was obtained from PubChem database (Table S1), (https://pubchem.ncbi.nlm.nih.gov/compound/Empagliflozin#section=InChIKey/, accessed on July 7, 2024) [9]. Five datasets were subsequently examined to identify the targets for empagliflozin (Table S2), as follows: (1) SwissTargetPrediction database (http://www.swisstargetprediction.ch/result.php?job=854031954&organism=Homo_sapiens/, accessed on July 7, 2024) [10]; (2) SuperPred database (https://prediction.charite.de/subpages/target_result.php, accessed on July 7, 2024) [11]; (3) DrugBank (https://go.drugbank.com/drugs/DB09038, accessed on July 7, 2024) (Knox et al. 2024); (4) Comparative Toxicogenomics Database (CTD), https://ctdbase.org/detail.go?type=chem&acc=C570240, accessed on July 7, 2024) [12]; and (5) The targetnet database (http://targetnet.scbdd.com/calcnet/calc_text/, accessed on July 7, 2024) [13]. For further investigation, the targets obtained from the five databases were standardized into valid gene symbols using the UniProt database (https://www.uniprot.org/, accessed on July 7, 2024) (Consortium, 2023). Through using Jvenn tool (https://jvenn.toulouse.inrae.fr/app/example.html, accessed on July 7, 2024) [14], all duplicate targets were removed.

#### 2.1.2 Identification of Targets Associated With Lung Cancer

The following keyword “lung cancer or lung neoplasm” was used to retrieve the molecular targets linked to the target diseases from five databases (Table S3): (1) GeneCards database (https://www.genecards.org/Search/Keyword?queryString=lung%20cancer, accessed on July 7, 2024) [15]; (2) Therapeutic Target Database (TTD, https://db.idrblab.net/ttd/search/ttd/disease?search_api_fulltext=lung%20cancer&page=0, accessed on July 7, 2024) [16]; (3) The Open Targets Platform database, https://platform.opentargets.org/disease/MONDO_0021117/associations, accessed on July 7, 2024) [17]; (4) Comparative Toxicogenomics Database (CTD), https://ctdbase.org/detail.go?type=disease&acc=MESH%3AD008175, accessed on July 7, 2024) [12]; and (5) MalaCards database (https://www.malacards.org/card/lung_cancer#Genes, accessed on July 7, 2024) [18]. The five databases provide supplementary resources for acquiring targets applicable to illnesses. All targets were converted into the uniform gene symbol format using UniProt and any duplicate targets were eliminated.

Because unfiltered keyword retrieval returns nearly the entire protein‐coding genome (29,425 unique symbols, approximately 148% of the ~19,900 human protein‐coding genes, reflecting the inclusion of non‐coding genes, pseudogenes and alias spellings), quantitative cutoffs were applied. Targets were retained if they carried an Open Targets Platform overall association score ≥ 0.30 for lung neoplasm (MONDO_0021117), retrieved through the Platform GraphQL API; all targets meeting this threshold are supported by at least one non‐text‐mining evidence class (genetic association, somatic mutation, clinical, affected pathway or animal model). GeneCards entries were restricted by relevance score, and TTD and MalaCards entries were retained as curated disease‐gene sets. Gene symbols from all sources were harmonized to current approved symbols before intersection.

#### 2.1.3 Construction of Disease‐Drug Common Targets, Protein–Protein Interaction, and Drug‐Target Networks

The empirical targets of empagliflozin and lung cancer were inputted into an online Jvenn software to create a Venn diagram illustrating the shared targets between them. The protein–protein interactions (PPI) network of the intersected targets was constructed by uploading the relevant targets to the STRING database Version 12.0 (https://string-db.org/, accessed on July 8, 2024) [19] with a default confidence level of > 0.4. The organism selected for the analysis was Homo sapiens. After constructing the PPI network, a compound‐target network was created to link empagliflozin with many overlapping targets.

The network visualization was performed using the Cytoscape 3.10.2 software developed by NIGMS in the United States [20]. Then, the common overlapped targets were filtered by degree value and subsequently, ranked based on Betweenness and Closeness by using Cytoscape plugin CytoNCA (Tang et al. 2014). The top 10‐ranked proteins were identified as hub targets.

#### 2.1.4 Functional Enrichment Analysis

The Gene Ontology (GO) analysis and Kyoto Encyclopedia of Genes and Genomes (KEGGs) pathway enrichments were conducted using the ShinyGO software (http://bioinformatics.sdstate.edu/go/#tab-6415-3, accessed on July 8, 2024) [21]. A critical P value of less than 0.05 was used as the cutoff value. The results were presented in the form of enrichment bubble for GO terms and Sankey and dot for KEGG pathway provided by SRplot website (https://www.bioinformatics.com.cn/en?keywords=ENRICHMENT, accessed on July 8, 2024) [22].

#### 2.1.5 Molecular Docking

Molecular docking was performed as described by Torres et al. [23]. The EMPA 3D structural model was obtained in SDF format from PubChem (CID 11949646) and converted to PDB format with Open Babel (version 2.4.1). Three target structures were retrieved from the RCSB Protein Data Bank: annexin A8 (ANXA8; UniProt P13928; PDB 1W3W, X‐ray, 1.99 Å, covering residues 1–327 and all four annexin repeats); the ZZ‐type zinc‐finger domain of p62/SQSTM1 (UniProt Q13501; PDB 5YP7, X‐ray, 1.424 Å, residues 126–180, covering 48 of the 51 annotated ZZ residues); and the ligand‐binding domain of PPARγ in complex with a PGC‐1α coactivator peptide (PPARγ, UniProt P37231, residues 234–505; PGC‐1α, UniProt Q9UBK2, residues 136–154; PDB 8BF1, X‐ray, 1.36 Å). Because PGC‐1α is present in 8BF1 only as a 19‐residue coactivator peptide, and because no deposited structure resolves a ligand‐binding pocket of PGC‐1α itself (the protein appears in all 48 of its PDB entries as an NR‐box peptide of at most 21 residues, 2.6% of its 798 residues), docking against this entry is attributed to PPARγ rather than to PPARGC1A. After removal of ligands and water, polar hydrogens were added in PyMOL 3.0, and the EMPA and target files were docked in PyRx (version 0.8); the nine highest‐ranked modes for each target, with binding energies in kcal/mol and lower‐ and upper‐bound RMSD values, are reported in Table S4, and interactions were visualized in BIOVIA Discovery Studio 2024.

To provide an independent check with fully documented search parameters, all docking was additionally repeated with AutoDock Vina 1.2.5. EMPA was prepared with Meeko 0.8.0 and RDKit, retaining the deposited stereochemistry and treating all 10 rotatable bonds as flexible, and receptors were prepared as rigid, protein‐only structures with waters, ions and other heteroatoms removed and polar hydrogens added; because the Vina scoring function has no parameters for coordinated metals, the structural Zn^2+^ ions of the p62 ZZ domain were not retained. Grid spacing was the Vina default of 0.375 Å and 20 modes were requested per run. As none of the three targets has a co‐crystallized drug‐like ligand from which to define a search box, a two‐stage protocol was used: a blind box enveloping the whole deposited chain or chains to locate the best‐scoring site (exhaustiveness 32), followed by a focused 22.5 Å cubic box centered on that site (exhaustiveness 64, three independent random seeds). Centers of all focused boxes are given in Table S5. The p62 ZZ domain was run both as the single chain A and as the two chains present in the deposited asymmetric unit (A and D).

The protocol was validated by re‐docking erlotinib into its crystallographic site in the EGFR kinase domain (PDB 1M17, ligand AQ4; box center 21.697, 0.303, 52.093 Å, size 25.7 × 14.7 × 17.1 Å; exhaustiveness 32): the top‐ranked pose reproduced the crystal pose with a symmetry‐corrected heavy‐atom RMSD of 1.30 ± 0.02 Å across three seeds (RDKit GetBestRMS), within the conventional 2.0 Å acceptance criterion. EMPA was additionally docked into the same validated EGFR site as a reference‐ligand comparison. Poses and contact residues were analyzed with RDKit; complete run parameters and all output modes are given in Tables S5 and S6. Docking scores are reported as geometric plausibility estimates and are not interpreted as evidence of direct binding or of binding affinity.

### 2.2 In‐Vitro Analysis

#### 2.2.1 Cell Lines and Culture Conditions

Two human lung cancer cell lines, **A549** and **HOP‐92**, were used to evaluate the antiproliferative, apoptotic, and autophagy‐modulating effects of the tested compounds. **A549**, a human lung adenocarcinoma cell line (ATCC® CCL‐185™), and **HOP‐92** (ATCC® CCL‐185™), a human non‐small‐cell lung cancer cell line, were obtained from the **Tissue Culture Unit of the Holding Company for Biological Products and Vaccines (VACSERA), Giza, Egypt**.

Cells were maintained in **RPMI‐1640 medium** supplemented with **10% fetal bovine serum (FBS)** and **1% penicillin–streptomycin**. Cultures were incubated at **37°C in a humidified atmosphere containing 5% CO_2_** and routinely monitored until approximately **70%–80% confluence** was reached. Cells were subsequently harvested and seeded at appropriate densities according to the requirements of each experimental assay. Cell culture procedures were performed under aseptic conditions using standard mammalian cell culture techniques [24].

For subsequent experiments, both cell lines were used to investigate the effects of **empagliflozin (EMPA)**, **hydroxychloroquine (HCQ)**, and their combination on cell viability, apoptosis, and autophagy‐related signaling [24].

#### 2.2.2 Cytotoxicity and Cell Viability Assay (MTT Assay)

The cytotoxic effects of empagliflozin (EMPA), HCQ, and their combination were evaluated in human lung cancer A549 and HOP‐92 cell lines using the 3‐(4,5‐dimethylthiazol‐2‐yl)‐2,5‐diphenyltetrazolium bromide (MTT) assay. Cells were exposed to five concentrations of each treatment (0.4, 1.6, 6.3, 25, and 100 µg/mL), and measurements were performed in triplicate. For combination treatment, EMPA and HCQ were administered at an equal‐dose ratio [24].

Briefly, A549 cells and HOP‐92 were seeded into 96‐well plates at a density of 1 × 10^5^ cells/mL (100 µL/well) and allowed to adhere for 24 h in complete RPMI‐1640 medium supplemented with 10% FBS and 1% penicillin–streptomycin at 37°C in 5% CO_2_. After incubation, the medium was replaced with fresh medium containing serial two‐fold dilutions of each drug (1000–31.25 µg/mL). Three wells containing only maintenance medium were used as untreated controls.

After a 48‐h treatment, 20 µL of MTT solution (5 mg/mL in PBS) was added to each well, and the plates were incubated for 4 h at 37°C to allow for the formation of insoluble formazan crystals. The supernatant was gently discarded, and the formazan crystals were dissolved in 200 µL DMSO. The absorbance was measured at 560 nm with a reference wavelength of 620 nm using a microplate reader.

The **percentage of cell viability** was calculated using the formula:

$$\text{Cell Viability}(\%)=\frac{{\text{OD}}_{\text{treated}}}{{\text{OD}}_{\text{control}}}\times 100$$

The **percentage of cytotoxicity** was obtained by subtracting the viability percentage from 100%. The **half‐maximal inhibitory concentration (IC_50_)**, representing the concentration required to reduce cell viability by 50%, was determined by nonlinear regression analysis of the dose–response curves using Microsoft Excel and GraphPad Prism software. The reported IC_50_ values were subsequently used for drug‐interaction analyses.

#### 2.2.3 Drug Combination Analysis

The interaction between EMPA and HCQ was evaluated using complementary dose‐additivity and response‐independence approaches. Combination index (CI) analysis was used to evaluate the interaction at the 50% effect level, whereas Bliss independence analysis was used to characterize the interaction across the tested concentration range. Because different reference models make different assumptions regarding non‐interaction, their results were interpreted as complementary rather than interchangeable measures of drug interaction [25] (Lederer et al. 2019).

### 2.3 Combination Index

The drug interaction at the 50% effect level was assessed using a Loewe dose‐additivity–based combination index (CI_50_). The CI concept provides a quantitative framework in which values below, equal to, or above 1 indicate synergistic, additive, or antagonistic interactions, respectively [25].

For the equal‐dose EMPA–HCQ combination, CI_50_ was calculated as:

$$CI_{50}=\frac{D_{\text{EMPA}}}{IC_{50,\text{EMPA}}}+\frac{D_{\text{HCQ}}}{IC_{50,\text{HCQ}}}$$

where $D_{\text{EMPA}}$ and $D_{\text{HCQ}}$ are the concentrations of EMPA and HCQ in the combination producing 50% inhibition, respectively, and $IC_{50,\text{EMPA}}$ and $IC_{50,\text{HCQ}}$ are the corresponding concentrations of the individual agents required to produce the same effect. Because EMPA and HCQ were combined at an equal‐dose ratio, the concentration of each compound at the combination IC_50_ was considered equivalent.

CI_50_ values were interpreted as follows: CI < 1, synergism; CI = 1, additivity; and CI > 1, antagonism [25].

### 2.4 Dose‐Reduction Index

The dose‐reduction index (DRI) was calculated to estimate the magnitude by which the concentration of each drug could be reduced when administered in combination while maintaining the same 50% inhibitory effect. DRI is commonly used together with CI analysis to describe the potential dose‐reduction advantage of drug combinations [25].

DRI was calculated as:

$$\mathrm{DRI}=\frac{D_{x}}{D_{c}}$$

where $D_{x}$ represents the concentration of the individual drug required to achieve 50% inhibition and $D_{c}$represents the concentration of the same drug in the combination required to produce the equivalent effect. A DRI > 1 indicates a favorable dose reduction.

### 2.5 Bliss Independence Analysis

The EMPA–HCQ interaction was additionally evaluated using the Bliss independence model at 0.4, 1.6, 6.3, 25, and 100 µg/mL. The Bliss model assumes independent action of the two agents and predicts the combined response from the responses produced by the individual treatments (Lederer et al. 2019).

For each concentration, the expected fractional viability under Bliss independence was calculated as:

$$V_{\text{Bliss}}=V_{\text{EMPA}}\times V_{\text{HCQ}}$$

where viability was expressed as a fraction of untreated control. Accordingly, the expected fractional effect was calculated as:

$Fa_{\text{expected}}=1-V_{\text{Bliss}}$

while the experimentally observed fractional effect of the combination was:

$Fa_{\text{observed}}=1-V_{\text{combination}}$

The excess‐over‐Bliss value was subsequently calculated as:

$\text{Bliss}\;\text{excess}\;=Fa_{\text{observed}}-Fa_{\text{expected}}$

Positive values indicate that the observed inhibitory effect exceeds that predicted under Bliss independence, values approaching zero indicate an approximately independent interaction, and negative values indicate an observed effect lower than the Bliss expectation.

#### 2.5.1 Cell Cycle Analysis

Cell‐cycle distribution was evaluated in **A549 and HOP‐92 human lung cancer cells** using the **Propidium Iodide Flow Cytometry Kit for Cell Cycle Analysis (ab139418, Abcam)** according to the manufacturer's instructions. The assay quantifies cellular DNA content using propidium iodide (PI) in combination with RNase [26].

Following treatment with **empagliflozin (EMPA)**, A549 and HOP‐92 cells were harvested as single‐cell suspensions, washed with PBS, and centrifuged at **500 × g for 5 min**. Cells were fixed in **66% ice‐cold ethanol** at 4°C for at least 2 h. Fixed cells were washed and resuspended in **200 µL PI/RNase staining solution**, followed by incubation at **37°C for 20–30 min in the dark**.

Samples were analyzed by flow cytometry using **488‐nm excitation**, with PI fluorescence collected in the **FL2 channel**. Appropriate FSC/SSC gating was applied to exclude debris and cell aggregates, and the percentages of cells in the **G0/G1, S, and G2/M phases** were determined.

#### 2.5.2 Annexin V‐FITC/PI Apoptosis Assay

Apoptosis and necrosis in **A549 and HOP‐92 human lung cancer cells** following EMPA treatment were evaluated using the **Annexin V‐FITC Apoptosis Detection Kit (BioVision, K101)** according to the manufacturer's protocol.

Approximately **1–5 × 10^5^ cells** were collected and resuspended in **500 µL of 1× binding buffer**. Subsequently, **5 µL Annexin V‐FITC and 5 µL PI** were added, and samples were incubated for **5 min at room temperature in the dark**. Samples were analyzed by flow cytometry at **488‐nm excitation**, with Annexin V‐FITC and PI fluorescence detected in the **FL1 and FL2 channels**, respectively [27].

Cells were classified as **early apoptotic, late apoptotic, or necrotic**, and the total cell‐death population was determined. Experiments were performed in **triplicate (*n*** = **3)**, and results were expressed as **mean ± SD**.

#### 2.5.3 Quantitative Real‐Time PCR (qPCR)

Total RNA was extracted using TRIzol™ Reagent (15596026; Life Technologies, USA) following the manufacturer's protocol [28]. After lysis with 1.2 mL TRIzol, samples were mixed with 0.2 mL chloroform/mL reagent, vortexed, and centrifuged at 12,000 × g for 15 min at 4°C. The aqueous phase containing RNA was precipitated with isopropanol, washed with 75% ethanol, and dissolved in RNase‐free water. RNA purity and concentration were measured spectrophotometrically at A_260_/A_280_, with ratios > 1.8 considered acceptable.

One microgram (1 µg) of total RNA was reverse‐transcribed using the **QuantiTect Reverse Transcription Kit (Qiagen, USA)**, incorporating **gDNA Wipeout Buffer** to remove genomic DNA. Reverse transcription was performed at **42°C for 15 min**, followed by enzyme inactivation at **95°C for 3 min**. cDNA was stored at **−20°C** for downstream analysis [28, 29].

Quantitative PCR was performed on a **Rotor‐Gene Q system (Qiagen, USA)** using **Maxima SYBR Green/Fluorescein qPCR Master Mix (Thermo Scientific)**. Each reaction (25 µL) contained 30 ng cDNA, 300 nM primers, and master mix. β‐actin was used as the housekeeping gene. The cycling program was: 95°C for 10 min, then 45 cycles of 95°C for 10 s, 60°C for 15 s, and 72°C for 15 s; followed by melting‐curve analysis from 72°C to 95°C (1°C/s) (Lee et al. 2019; [29]). **Primer sequences illustrated in** Table 1.

**TABLE 1:** Primer sequences.

Gene	Primer Sequences (5′–3′)	GenBank Accession No	Reference
BAX	F: TCAGGATGCGTCCACCAAGAAG R: TGTGTCCACGGCGGCAATCATC	NM_138761.5	OriGene
CASP3	F: GGAAGCGAATCAATGGACTCTGG R: GCATCGACATCTGTACCAGACC	NM_004346.4	OriGene
GAPDH	F: GTCTCCTCTGACTTCAACAGCG R: ACCACCCTGTTGCTGTAGCCAA	NM_002046.7	OriGene
P62	F: GTACCAGGACAGCGAGAGGAA R: CCCATGTTGCACGCCAAAC	NM_003900.6	[30]

### 2.6 Relative Gene Expression

Relative mRNA expression of **BAX**, **CASP3**, and **P62** was analyzed using the **2^−^ΔΔCt method** [29]. The **ΔCt** value was obtained by normalizing the Ct of the target gene to that of **β‐actin**, and **ΔΔCt** was calculated relative to the untreated control. Fold‐change expression was computed as **2^−^ΔΔCt**.

### 2.7 Assessment of Cleaved Caspase‐3 Activation by Flow Cytometry

Cleaved caspase‐3 was assessed by intracellular flow cytometry using the BD Pharmingen™ FITC Active Caspase‐3 Apoptosis Kit (Cat. No. 550480, BD Biosciences) according to the manufacturer's instructions [31]. The kit contains a FITC‐conjugated rabbit monoclonal antibody (clone C92‐605) that specifically recognizes the active form of caspase‐3 without recognizing the inactive procaspase‐3 form.

Following the designated experimental treatments, A549 and HOP‐92 cells were harvested and washed twice with cold 1× phosphate‐buffered saline (PBS). Cells were then resuspended in BD Cytofix/Cytoperm™ fixation and permeabilization solution at a density of approximately 1 × 10^6^ cells/0.5 mL and incubated for 20 min on ice. After fixation and permeabilization, the cells were pelleted and washed twice with 1× BD Perm/Wash™ buffer.

For intracellular staining, approximately 1 × 10^6^ cells were resuspended in 100 µL of 1× BD Perm/Wash™ buffer, followed by the addition of 20 µL FITC‐conjugated anti‐active caspase‐3 antibody. Samples were incubated for 30 min at room temperature, protected from light. Subsequently, cells were washed with 1 mL of 1× BD Perm/Wash™ buffer and finally resuspended in 0.5 mL of 1× BD Perm/Wash™ buffer for flow cytometric analysis.

Samples were analyzed by flow cytometry, and the percentage of active/cleaved caspase‐3‐positive cells was determined from FITC fluorescence histograms. Cells within the low‐fluorescence region (M1) were considered cleaved caspase‐3‐negative, whereas cells exhibiting increased FITC fluorescence within the M2 region were considered cleaved caspase‐3‐positive. This gating interpretation is consistent with the manufacturer's representative analysis, in which apoptotic cells shift into the active caspase‐3‐positive M2 population.

### 2.8 Western Blot Analysis of LC3‐II and p62/SQSTM1

The protein expression levels of the autophagy‐related markers microtubule‐associated protein 1 light chain 3 beta (LC3B/LC3‐II) and sequestosome 1 (SQSTM1/p62) were evaluated by Western blotting in A549 and HOP‐92 lung cancer cells, following standard immunoblotting procedures with modifications appropriate for autophagy assessment [5]. Cells were divided into control, empagliflozin (EMPA), hydroxychloroquine sulfate (HCQ), and EMPA + HCQ groups. Cells were seeded in 6‐well plates at approximately 1 × 10^5^–2 × 10^5^ cells/well and cultured until reaching 70%–80% confluence, as specified in the experimental protocol.

Following treatment, cells were washed twice with ice‐cold phosphate‐buffered saline (PBS) and lysed using 100–150 µL of pre‐chilled RIPA lysis buffer supplemented with protease inhibitor cocktail. Cell lysates were incubated on ice for 30 min with intermittent vortexing and subsequently centrifuged at 13,000 rpm for 10 min at 4°C. The resulting supernatants were collected, and total protein concentrations were determined using the bicinchoninic acid (BCA) protein assay with bovine serum albumin as the standard.

Protein samples were mixed with 4× Laemmli sample buffer, denatured at 95°C–100°C for 5–10 min, and subjected to sodium dodecyl sulfate‐polyacrylamide gel electrophoresis (SDS‐PAGE). A 15% polyacrylamide gel was used to facilitate resolution of the low‐molecular‐weight LC3 proteins. Proteins were subsequently transferred onto 0.22‐µm PVDF membranes by wet transfer at 100 V for 60–90 min under cold conditions. The use of a high‐percentage gel and small‐pore membrane is particularly important for efficient detection and retention of LC3‐II [5]. The detailed electrophoresis and transfer conditions used in the study are provided in the experimental protocol.

Following transfer, membranes were blocked with 5% non‐fat dry milk or 5% bovine serum albumin in TBST for 1 h at room temperature and then incubated overnight at 4°C with primary antibodies against LC3B, p62/SQSTM1, and β‐actin. The specified antibodies included anti‐LC3B (Abcam, ab192890), anti‐SQSTM1/p62 (Abcam, ab280086), and anti‐β‐actin (Abcam, ab13822), at a dilution of 1:1000. LC3‐II was detected at approximately 14–16 kDa, p62 at approximately 62 kDa, and β‐actin at approximately 40–42 kDa.

After washing, membranes were incubated with the appropriate horseradish peroxidase (HRP)‐conjugated secondary antibodies (1:5000–1:10,000) for 1 h at room temperature. Immunoreactive bands were visualized using an enhanced chemiluminescence (ECL) substrate and captured using a chemiluminescence imaging system.

Band intensities were quantified by densitometric analysis using ImageJ or Image Lab software. LC3‐II and p62 protein levels were normalized to β‐actin, which served as the internal loading control. Relative LC3‐II/β‐actin and p62/β‐actin expression levels were then determined for each experimental group.

LC3‐II and p62 were interpreted together rather than independently, because increased LC3‐II may reflect either enhanced autophagosome formation or impaired autophagosome degradation. Accordingly, HCQ, a lysosomal inhibitor, was included to assess autophagic flux. Accumulation of LC3‐II together with p62 following lysosomal inhibition was interpreted in the context of altered autophagic degradation [5, 32].

### 2.9 Statistical Analysis

Data were analyzed using GraphPad Prism version 9.0 (GraphPad Software Inc., San Diego, CA, USA). The normality of data distribution was verified using the Shapiro–Wilk test. Differences among treatment groups were evaluated using one‐way analysis of variance (ANOVA), followed by Tukey's multiple comparison post hoc test to identify specific intergroup differences. All experimental data were expressed as mean ± standard deviation of the mean (SD). The statistical significance level was established at *p* < 0.05.

## 3 Results

### 3.1 The Network Pharmacology Analysis

#### 3.1.1 Identifying the Targets Associated With Empagliflozin

In order to identify the targets linked to empagliflozin, we analyzed five databases. A total of 268 targets were found, with 100 sourced from The SwissTargetPrediction database, 130 from SuperPred, 10 from DrugBank, 11 from Comparative Toxicogenomics Database, and 17 from TargetNet database. Following the elimination of duplicates, the number of targets decreased to 246 (Table S7).

#### 3.1.2 Identifying the Targets Associated With Lung Cancer

In addition, a comprehensive analysis of five databases was conducted to determine the targets associated with lung cancer. A total of 55,328 targets were identified, 26,719 obtained from The GeneCards database, 12,995 from OpenTargets Platform database, 121 from TTD: Therapeutic Target Database, 14,669 from Comparative Toxicogenomics Database, and 824 from MalaCards databases. After the removal of duplicate entries, the total number of targets reduced to 29,540 (Table S8).

Re‐reading Table S8 gives 29,425 unique gene symbols, approximately 148% of the ~19,900 human protein‐coding genes. Applying an Open Targets overall association‐score cutoff of ≥ 0.30 to the current release (16,501 targets with a non‐zero association to lung neoplasm, of which 12,476, or 75.6%, rest on text‐mining evidence alone) retained 846 targets; cutoffs of ≥ 0.10 and ≥ 0.50 retained 2452 and 283 targets, respectively (Table S9, Figure 1, 3).

**FIGURE 1:**
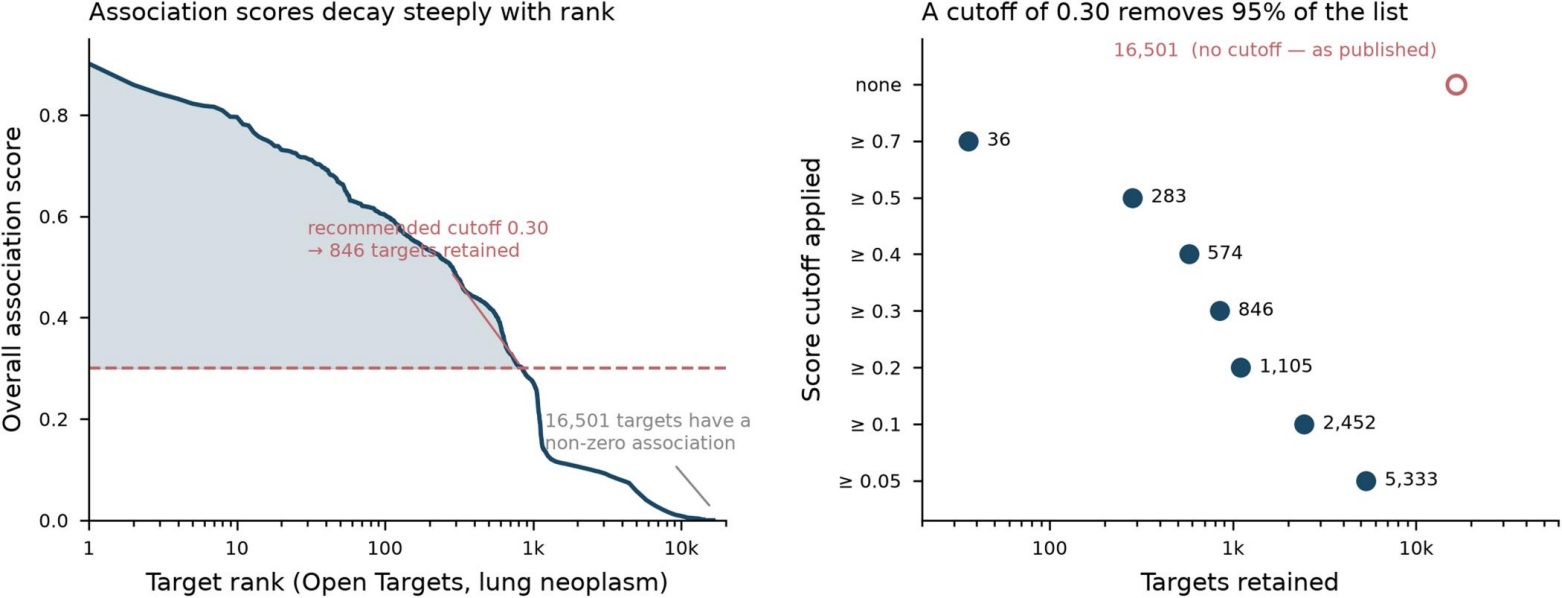
(A) Effect of an association‐score cutoff on the size of the lung‐cancer target list. Left panel: Open Targets Platform overall association scores for lung neoplasm (MONDO_0021117) plotted against target rank (logarithmic axis). Scores decline steeply with rank, and 16,501 targets carry a non‐zero association. The dashed line marks the applied cutoff of 0.30, which retains 846 targets (shaded area). Right panel: number of targets retained at each score cutoff (logarithmic axis); cutoffs of ≥ 0.05, ≥ 0.1, ≥ 0.2, ≥ 0.3, ≥ 0.4, ≥ 0.5 and ≥ 0.7 retain 5333, 2452, 1105, 846, 574, 283 and 36 targets, respectively, compared with 16,501 targets when no cutoff is applied (open circle), as in the originally submitted analysis. The 0.30 cutoff therefore removes approximately 95% of the unfiltered list. Scores were retrieved from the current Open Targets release through the Platform GraphQL API (Table S9).

#### 3.1.3 Targets of Empagliflozin Overlapped With Lung Cancer

Among the 240 unique targets for empagliflozin and the 29,425 unique targets for lung cancer, 239 targets were shared (Table S10, Figure 1, 3). Because the unfiltered disease list covers almost the entire protein‐coding genome, this intersection retained 99.6% of the drug‐target list and excluded only one gene (GBA); it therefore did not act as a disease filter. After the association‐score cutoff of ≥ 0.30 was applied to the disease side, 46 of the 239 targets were retained, an 80.8% reduction (Table S9). Six of the 239 have no recorded lung‐cancer association at any score.

#### 3.1.4 Protein–Protein Interaction (PPI) Network of the 239 Drug‐Diseases Overlapped Targets

A PPI diagram was generated by uploading all 239 overlapped targets to the STRING database to visualize the interactions among them. After excluding the unconnected nodes, the PPI network consisted of 237 nodes and 1707 edges (Figure 1, 3). The Cytoscape software 3.10.2 was used to integrate this network for additional analysis. The CytoNCA app was used to rank the 128 targets based on Betweenness and Closeness after they were filtered by the degree value (Table S10). Then, the 128 common targets were input into Cytoscape to construct a drug‐targets network (Figure 1, 3). In addition, a PPI network of the top 10 hub targets (GAPDH, IL1B, EGFR, HSP90AA1, NFKB1, HSP90AB1, MAPK3, MTOR, MAPK1, and EZH2) with the experimentally validated targets (ANXA8, BAX, CASP3, SQSTM1, MAP1LC3B, PPARG, and PPARGC1A) was created (Figure 1, 3). Of these 17 targets, nine carry an Open Targets association score ≥ 0.30 for lung neoplasm (Figure S1).

#### 3.1.5 Enrichment Analysis of the Related Proteins

The 128 overlapping targets were submitted to the ShinyGO analysis tool for GO enrichment analysis and KEGG Pathways in order to validate the important functional and biological properties of the 128‐relevant proteins. Statistical enrichment analysis was conducted in biological processes (BP), cellular components (CC), molecular functions (MF), and KEGG Pathways with a *p* value < 0.05 (Table S11, Table 2; Figure 1, 3). Top BP included cellular response to chemical stimulus, response to organonitrogen compound, response to nitrogen compound, and response to organic substance; Top CC included cell surface, membrane microdomain, membrane raft, plasma membrane region, and cell junction; Top MF included small molecule binding, nucleoside phosphate binding, nucleotide binding, and anion binding.

**TABLE 2:** Molecular docking results and search parameters.

Target (PDB, chains)	Role	Reported (kcal/mol)	Re‐docked (kcal/mol)	Box center x, y, z (Å)	Exhaust.	RMSD (Å)
EGFR (1M17, A) + erlotinib	protocol validation	—	−7.37 ± 0.03	21.697, 0.303, 52.093	32	1.30 ± 0.02
EGFR (1M17, A) + empagliflozin	reference‐ligand control	—	−9.01 ± 0.03	21.697, 0.303, 52.093	32	n.a.
ANXA8 (1W3W, A)	target	−7.6	−7.63 ± 0.01	4.753, 5.987, 1.137	64	n.a.
p62/SQSTM1 ZZ (5YP7, A + D)	target	−7.0	−6.96 ± 0.00	139.756, 171.362, 234.739	64	n.a.
p62/SQSTM1 ZZ (5YP7, A only)	single‐chain comparison	—	−5.58 ± 0.00	144.628, 162.396, 244.838	64	n.a.
PPARγ (8BF1, A + B)	target, rebelled from PPARGC1A	−7.5	−8.30 ± 0.02	47.237, 7.548, 1.799	64	n.a.

*Note:* Reported score is the top‐ranked mode of the original PyRx run (Table S4). Re‐docked score is the mean ± SD of three independent seeds in the focused box with AutoDock Vina 1.2.5 (grid spacing 0.375 Å, 20 modes requested per run, focused box 22.5 × 22.5 × 22.5 Å except the two EGFR runs, which used a 25.7 × 14.7 × 17.1 Å box centered on the erlotinib crystal pose). RMSD is the symmetry‐corrected heavy‐atom deviation of the top‐ranked pose from the crystallographic pose. n.a., not applicable (no co‐crystallized ligand for that target); —, not part of the original analysis.

To determine the likely pathways associated with the effect of EMPA against lung cancer, a KEGG pathway analysis was performed. This analysis included 128 relevant targets with a P value level set < 0.05. The pathways in cancer and Kaposi sarcoma‐associated herpesvirus infection were identified among the top 30 KEGG Pathways involved in the pathophysiology of lung cancer (Table S11, Figure 1, 3). Significantly, these pathways were also found to be associated with some of the experimentally validated targets, namely BAX, CASP3, MAP1LC3B, and PPARG (Figure 1, 3).

#### 3.1.6 Molecular Docking of Empagliflozin Against Structurally Validated Targets

Docking was performed against annexin A8 (PDB 1W3W), the ZZ‐type zinc‐finger domain of p62/SQSTM1 (PDB 5YP7) and the PPARγ ligand‐binding domain in complex with a PGC‐1α coactivator peptide (PDB 8BF1). The highest‐ranked mode for each target gave binding energies of −7.6, −7.0 and −7.5 kcal/mol, respectively (Table S4, Table 3; Figure 2A,B). Because PGC‐1α is represented in 8BF1 only by a 19‐residue coactivator peptide, the third value is attributed to PPARGC1A; PPARG is itself among the targets examined experimentally here and is retained by the association‐score cutoff (score 0.448, rank 366 of 16,501).

**TABLE 3:** Cytotoxic potency and interaction of empagliflozin and hydroxychloroquine in A549 and HOP‐92 cells.

Cell line	EMPA IC_50_ (µg/mL)	HCQ IC_50_ (µg/mL)	EMPA + HCQ IC_50_* (µg/mL)	CI_50_	DRI‐EMPA	DRI‐HCQ	Max. Bliss excess (dose)
A549	52.905 ± 1.85	58.532 ± 1.52	**11.286** ± **0.36**	**0.406**	**4.69**	**5.19**	**+0.080 (1.6 µg/mL)**
HOP‐92	81.629 ± 2.50	109.810 ± 3.70	**28.919** ± **1.08**	**0.618**	**2.82**	**3.80**	**+0.091 (6.3 µg/mL)**

*Note:* Values are presented as IC_50_ ± SD where available. EMPA, empagliflozin; HCQ, hydroxychloroquine; CI_50_, combination index at 50% effect; DRI, dose‐reduction index. The EMPA–HCQ combination was applied at a 1:1 equal‐dose ratio. CI_50_ < 1 indicates synergy under the Loewe dose‐additivity model, whereas DRI > 1 indicates favorable dose reduction. Positive Bliss excess indicates an observed inhibitory effect greater than that predicted by Bliss independence. CI_50_, DRI, and Bliss excess were calculated from the experimental MTT data.

**FIGURE 2:**
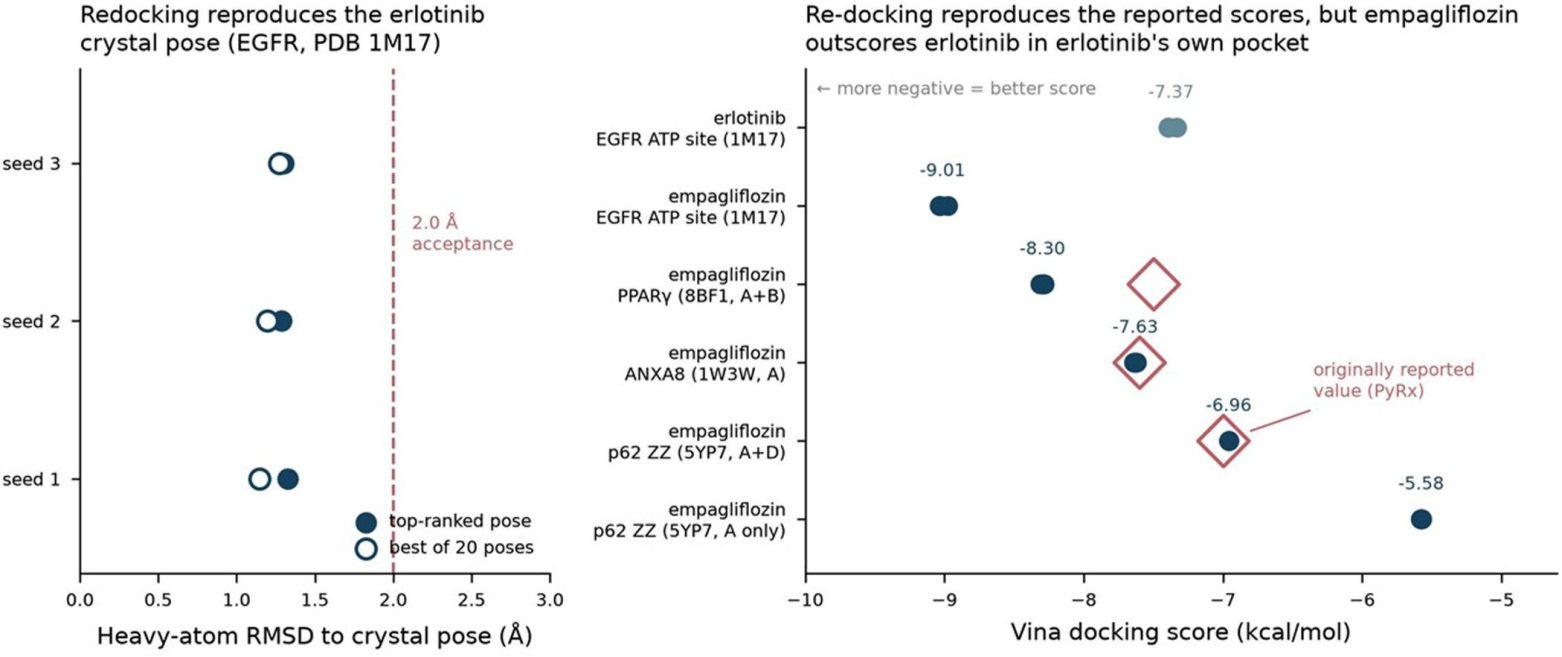
Validation of the docking protocol and re‐docking of empagliflozin with AutoDock Vina 1.2.5. (A, left panel) Re‐docking of erlotinib into its crystallographic site in the EGFR kinase domain (PDB 1M17) in three independent random seeds. Filled circles show the symmetry‐corrected heavy‐atom RMSD of the top‐ranked pose to the crystal pose, and open circles the lowest RMSD among the 20 output poses. All top‐ranked poses fall below the conventional 2.0 Å acceptance criterion (dashed line; 1.30 ± 0.02 Å). (B, right panel) Vina docking scores (kcal/mol; filled circles, individual seeds; numbers, mean of three seeds) for erlotinib and empagliflozin in the validated EGFR ATP site (1M17) and for empagliflozin against PPARγ (8BF1, chains A + B), ANXA8 (1W3W, chain A) and the p62/SQSTM1 ZZ domain (5YP7, chains A + D, and chain A alone). Open diamonds indicate the values originally reported with PyRx (Table S4); more negative values indicate more favorable scores. Empagliflozin scores more favorably than erlotinib in erlotinib's own crystallographic pocket despite having no reported EGFR activity, so the scores are interpreted as geometric plausibility estimates rather than evidence of binding. Search‐box centers and run parameters are given in Table 3 and Tables S5 and S6.

Independent re‐docking with AutoDock Vina 1.2.5, using a protocol validated by reproducing the crystallographic pose of erlotinib in the EGFR kinase domain (heavy‐atom RMSD 1.30 ± 0.02 Å over three seeds, within the conventional 2.0 Å acceptance criterion; Table 3, Figure 2A), reproduced these values. EMPA was accommodated in a shallow groove of ANXA8 formed at the interface of annexin repeats 2, 3 and 4 (−7.63 ± 0.01 kcal/mol; contacts Leu120–Lys125, Leu162, Ser166–Val170, Leu205–Leu213, Val247, Cys250, Thr251, Leu254). At the p62 ZZ domain the score depended on how much of the deposited structure was retained: −6.96 ± 0.00 kcal/mol using both chains of the asymmetric unit, with the best‐scoring site lying at the chain A–chain D interface (contacts Gly130, Cys131, Lys157–Gly162 in chain A and Pro134–Gly137, Cys151–Val153 in chain D), against −5.58 ± 0.00 kcal/mol for the isolated single chain. At PPARγ, EMPA scored −8.30 ± 0.02 kcal/mol with the pose occupying the Ω‐pocket and β‐sheet region of the ligand‐binding domain (19 contacts, including Cys285, Arg288, Ser342, Met348, Leu353 and Met364) and making no contact with the PGC‐1α peptide, confirming that this calculation characterizes the PPARγ pocket.

These values should be read as geometric plausibility estimates rather than as evidence of binding. In the validated EGFR site used as a positive control, EMPA scored −9.01 ± 0.03 kcal/mol, a more favorable value than erlotinib itself (−7.37 ± 0.03 kcal/mol), despite EMPA having no reported EGFR activity among its 79 ChEMBL activity records, whereas erlotinib has a median potency of 28.5 nM across 239 records (Figure 2B). Scores in this range for a 450‐Da ligand with 10 rotatable bonds therefore reflect ligand size and surface complementarity as much as target identity. Complete run parameters and all output modes are given in Tables S5 and S6, and the inventory of experimental structures available for the three targets in Table S12.

### 3.2 In‐Vitro Experimental Results

#### 3.2.1 Cytotoxic Effects of Empagliflozin and Hydroxychloroquine in A549 and HOP‐92 Cells

The cytotoxic effects of empagliflozin (EMPA), hydroxychloroquine (HCQ), and their combination were evaluated in **A549** and **HOP‐92** lung cancer cells using the MTT assay over a concentration range of 0.4–100 µg/mL. Both individual treatments produced concentration‐dependent reductions in cell viability, while combined EMPA and HCQ treatment resulted in greater cytotoxicity than either single agent across the tested concentration range.

**In A549 cells,** EMPA reduced cell viability from 94.41% at 0.4 µg/mL to 34.27% at 100 µg/mL, whereas HCQ reduced viability from 93.65% to 36.92%. Combined treatment produced a greater reduction in cell viability, from 83.44% at 0.4 µg/mL to 28.05% at 100 µg/mL. The enhancement was particularly evident at intermediate concentrations. At 6.3 µg/mL, cell viability was 76.92% following EMPA and 79.81% following HCQ treatment, compared with 53.72% following combined treatment. Similarly, at 25 µg/mL, viability was reduced to 42.65% by the combination compared with 65.69% and 63.13% following EMPA and HCQ monotherapies, respectively.

**HOP‐92 cells** also exhibited concentration‐dependent responses, although they were comparatively less sensitive to the individual treatments. EMPA reduced cell viability from 100.77% at 0.4 µg/mL to 39.13% at 100 µg/mL, while HCQ reduced viability from 100.10% to 41.48%. The EMPA–HCQ combination decreased viability from 98.86% to 32.35% over the same concentration range. At 6.3 µg/mL, combined treatment reduced viability to 70.34%, compared with 88.06% for EMPA and 90.26% for HCQ alone. At 25 µg/mL, the corresponding values were 52.14%, 67.04%, and 70.15%, respectively.

#### 3.2.2 Combination Treatment Markedly Reduced Ic_50_ Values

Analysis of half‐maximal inhibitory concentrations demonstrated increased cytotoxic potency following combined EMPA and HCQ treatment. **In A549 cells, the IC_50_ values** of EMPA and HCQ were 52.905 ± 1.85 and 58.532 ± 1.52 µg/mL, respectively. In contrast, the IC_50_ of the EMPA–HCQ combination was 11.286 ± 0.36 µg/mL. Thus, the concentration required to achieve 50% inhibition in the combination was approximately 4.69‐fold lower than the IC_50_ of EMPA alone and 5.19‐fold lower than that of HCQ alone (Table 3, Figure 1, 3).

**Figure 3:**
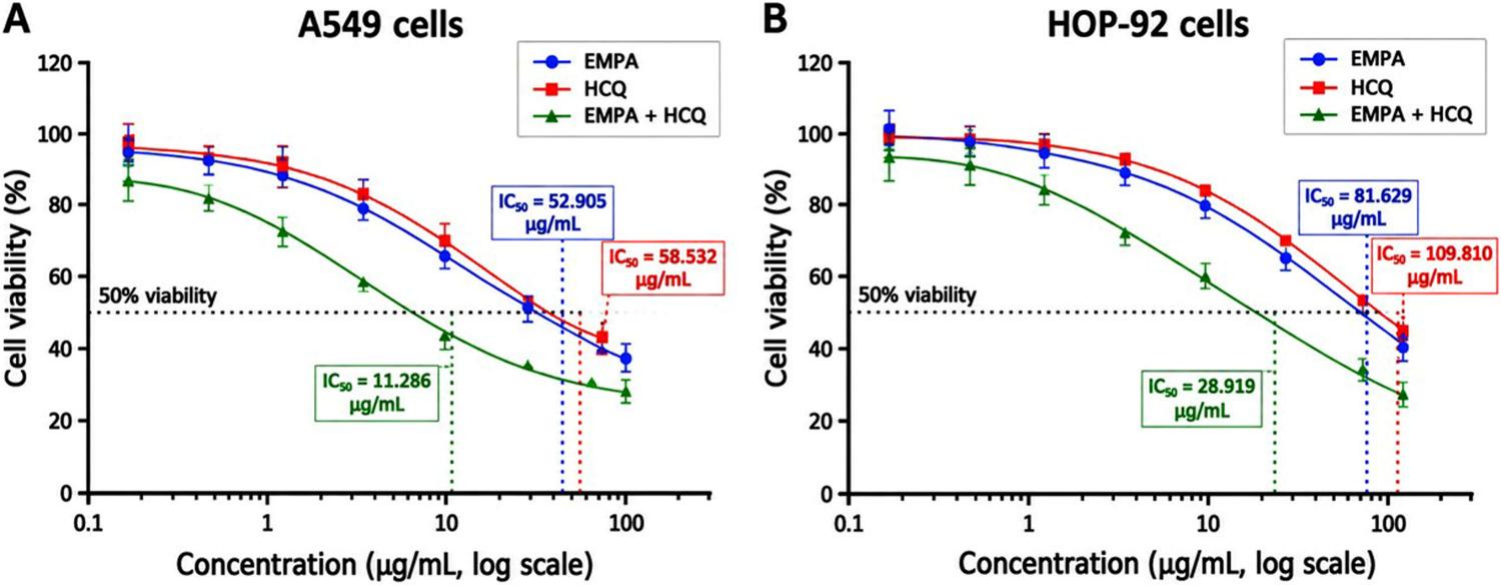
(A, B) Dose‐dependent cytotoxic effects of empagliflozin (EMPA), HCQ, and their combination in A549 and HOP‐92 cells. Cell viability was evaluated using the MTT assay following treatment with increasing concentrations (0.4–100 µg/mL) of EMPA, HCQ, or EMPA + HCQ. (A) A549 cells and (B) HOP‐92 cells. The concentration axis is presented on a logarithmic scale. The horizontal dashed line represents 50% cell viability, while the indicated IC_50_ values represent the concentrations required to reduce cell viability by 50%. In A549 cells, the IC_50_ values were 52.905, 58.532, and 11.286 µg/mL for EMPA, HCQ, and EMPA + HCQ, respectively. In HOP‐92 cells, the corresponding IC_50_ values were 81.629, 109.810, and 28.919 µg/mL. The marked reduction in IC_50_ following combined treatment indicates enhanced cytotoxic activity compared with either agent alone.

A similar enhancement was observed in **HOP‐92 cells.** The IC_50_ values of EMPA and HCQ were 81.629 ± 2.50 and 109.81 ± 3.70 µg/mL, respectively, whereas the combination exhibited an IC_50_ of 28.919 ± 1.08 µg/mL. This represented approximately 2.82‐ and 3.80‐fold reductions relative to the individual IC_50_ values of EMPA and HCQ, respectively. The greater reduction in IC_50_ observed in A549 cells indicated a more pronounced enhancement of combination potency in this cell line (Table 3, Figure 1, 3).

#### 3.2.3 Combination Index and Dose‐Reduction Analysis

The interaction between EMPA and HCQ was further evaluated at the 50% effect level using a Loewe dose‐additivity approach. As the two agents were combined at an equal‐dose ratio, the concentration of each compound at the combination IC_50_ was used to calculate the combination index (CI_50_).

In A549 cells, the calculated CI_50_ was 0.406, indicating a synergistic interaction at the 50% effect level. The corresponding dose‐reduction indices (DRIs) were 4.69 for EMPA and 5.19 for HCQ. Therefore, when used in combination, approximately 4.7‐fold and 5.2‐fold lower concentrations of EMPA and HCQ, respectively, were required to achieve a 50% inhibitory effect compared with their respective single‐agent IC_50_ values (Table 3, Figure 4).

**FIGURE 4:**
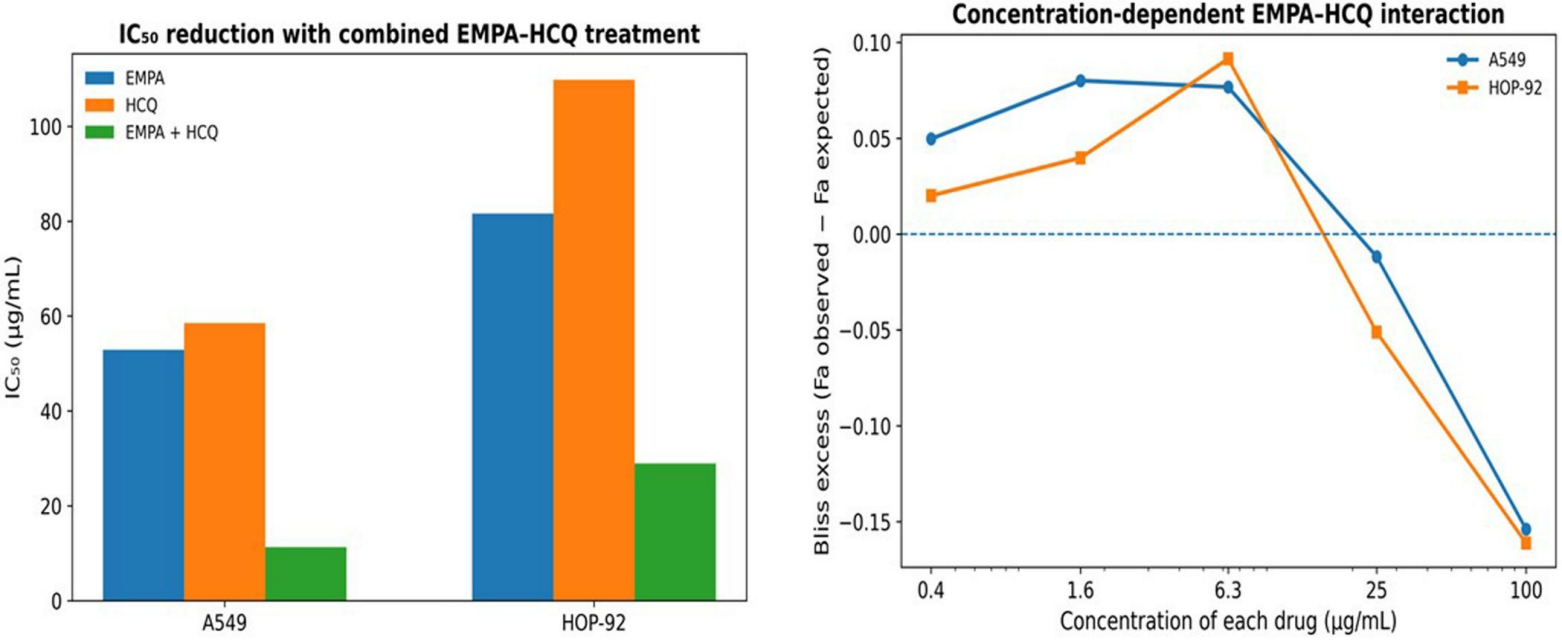
IC_50_ reduction and concentration‐dependent interaction of empagliflozin (EMPA) and HCQ in A549 and HOP‐92 cells. (A) Comparison of IC_50_ values for EMPA, HCQ, and the EMPA–HCQ combination. Combined treatment markedly reduced the IC_50_ compared with either single agent in both cell lines, with IC_50_ values of 11.286 µg/mL in A549 and 28.919 µg/mL in HOP‐92 cells. (B) Concentration‐dependent drug interaction assessed using the Bliss independence model. Bliss excess was calculated as the difference between the observed and Bliss‐expected fractional effects (Fa_observed − Fa_expected). Positive values indicate an inhibitory effect greater than predicted by Bliss independence, values around zero indicate an approximately independent/additive interaction, and negative values indicate an effect lower than the Bliss expectation. Positive Bliss excess was observed predominantly at low‐to‐intermediate concentrations, whereas negative values were observed at higher concentrations (25–100 µg/mL), demonstrating a concentration‐dependent EMPA–HCQ interaction.

In HOP‐92 cells, the CI_50_ was 0.618, also indicating synergy at the 50% effect level. The corresponding DRI values were 2.82 for EMPA and 3.80 for HCQ. Thus, the combination reduced the concentrations required for 50% inhibition by approximately 2.8‐fold for EMPA and 3.8‐fold for HCQ (Table 3, Figure 4).

Collectively, the CI_50_ and DRI analyses demonstrated synergistic dose interactions at the 50% effect level in both cell lines, with a lower CI_50_ and greater dose‐reduction effects in A549 cells.

#### 3.2.4 Concentration‐Dependent Interaction According to Bliss Independence Analysis

To further characterize the interaction across the complete concentration range, the observed combination responses were compared with the responses predicted by the Bliss independence model. Positive excess‐over‐Bliss values indicate that the experimentally observed inhibitory effect exceeded that expected from independent drug action.

In A549 cells, positive excess‐over‐Bliss values were observed at 0.4, 1.6, and 6.3 µg/mL, with values of approximately 0.050, 0.080, and 0.077, respectively. The greatest positive deviation from Bliss independence therefore occurred at 1.6 µg/mL. At 25 µg/mL, the observed combination effect was close to the Bliss prediction (excess‐over‐Bliss, −0.012), whereas at 100 µg/mL the observed effect was lower than the Bliss‐predicted effect (−0.154) (Table 3, Figure 4).

A similar concentration‐dependent pattern was observed in HOP‐92 cells. Positive excess‐over‐Bliss values of approximately 0.020, 0.040, and 0.091 were obtained at 0.4, 1.6, and 6.3 µg/mL, respectively, with the largest positive deviation occurring at 6.3 µg/mL. In contrast, excess‐over‐Bliss values became negative at 25 and 100 µg/mL (−0.051 and −0.161, respectively) (Table 3, Figure 4).

These findings demonstrate that the EMPA–HCQ interaction was dependent on concentration. Although the combination consistently produced greater cytotoxicity than either individual agent, enhancement beyond the Bliss‐independent expectation was predominantly observed at low‐to‐intermediate concentrations. In contrast, the highest concentrations did not exceed the cytotoxic effect predicted by the Bliss model.

Overall, EMPA and HCQ displayed concentration‐dependent cytotoxicity in A549 and HOP‐92 cells, while their combination substantially reduced the concentration required to achieve 50% inhibition. CI_50_ values of 0.406 and 0.618 in A549 and HOP‐92 cells, respectively, supported synergistic dose interactions at the 50% effect level, while Bliss analysis demonstrated that the magnitude of the interaction varied across the tested concentration range.

#### 3.2.5 Dose Selection Rationale for Empagliflozin

Based on the MTT cytotoxicity results, subsequent mechanistic experiments were performed using concentrations corresponding to **0.5× IC_50_ and 1× IC_50_** to investigate treatment‐induced molecular and cellular alterations while minimizing excessive nonspecific cytotoxicity. In A549 cells, the IC_50_ values of EMPA, HCQ, and their combination were **52.905** ± **1.85, 58.532** ± **1.52, and 11.286** ± **0.36 µg/mL**, respectively, whereas the corresponding values in HOP‐92 cells were **81.629** ± **2.50, 109.810** ± **3.70, and 28.919** ± **1.08 µg/mL**. Accordingly, the **0.5× IC_50_** concentration was selected as a sublethal exposure to permit detection of early treatment‐induced signaling, oxidative, mitochondrial, and apoptotic alterations while maintaining a sufficient population of viable cells. In contrast, the **1× IC_50_** concentration was selected to characterize established molecular and apoptotic responses under conditions producing approximately 50% inhibition of cell viability.

For **reactive oxygen species (ROS) generation** a **24‐h exposure** can be used to assess relatively early oxidative and mitochondrial alterations preceding extensive cell loss. Similarly, **caspase‐3 activation and apoptosis assays** can be performed after **24 h** to determine whether the cytotoxic response is associated with activation of the apoptotic machinery. For **Western blot analysis**, treatment for **24 h** can be used to evaluate early changes in the expression or activation of proteins involved in the proposed signaling and cell‐death pathways. In contrast, **cell‐cycle distribution can be assessed after 48 h**, providing sufficient time for treatment‐induced alterations in cell‐cycle progression and accumulation of cells within specific phases to become apparent.

#### 3.2.6 Effect of Empagliflozin on Cell‐Cycle Progression in A549 and HOP‐92 Cells

Flow‐cytometric analysis revealed that empagliflozin (EMPA) markedly altered cell‐cycle distribution in both A549 and HOP‐92 lung cancer cells. In A549 cells, EMPA increased the G0/G1 population from **58.03% in control cells to 72.02%**, accompanied by reductions in the S‐phase population from **34.16% to 22.69%** and the G2/M population from **7.81% to 5.29%**. These changes indicate an accumulation of A549 cells in G0/G1, consistent with the **G1 cell‐cycle arrest** reported in the flow‐cytometric analysis (Table 4, Figure 5).

**TABLE 4:** Effect of empagliflozin on cell‐cycle distribution in A549 and HOP‐92 cells.

Cell line	Treatment	G0/G1 (%)	S phase (%)	G2/M (%)	Cell‐cycle response
A549	Control	58.03 ± 4.43^b^	34.16 ± 3.43^a^	7.81 ± 3.43^a^	—
	EMPA	72.02 ± 5.43^a^	22.69 ± 2.43^b^	5.29 ± 1.43^a^	G1 arrest
HOP‐92	Control	53.19 ± 3.24^a^	29.55 ± 2.25^b^	17.26 ± 1.29^a^	—
	EMPA	62.08 ± 4.68^a^	35.15 ± 1.46^a^	2.77 ± 0.47^b^	G1/S arrest

*Note:* Data are expressed as mean ± SD (*n* = 3). Within each cell line and parameter, values bearing different superscript letters (a, b) are significantly different at *p* < 0.05, whereas values sharing the same letter are not significantly different, as determined using a two‐tailed Welch's *t*‐test.

**FIGURE 5:**
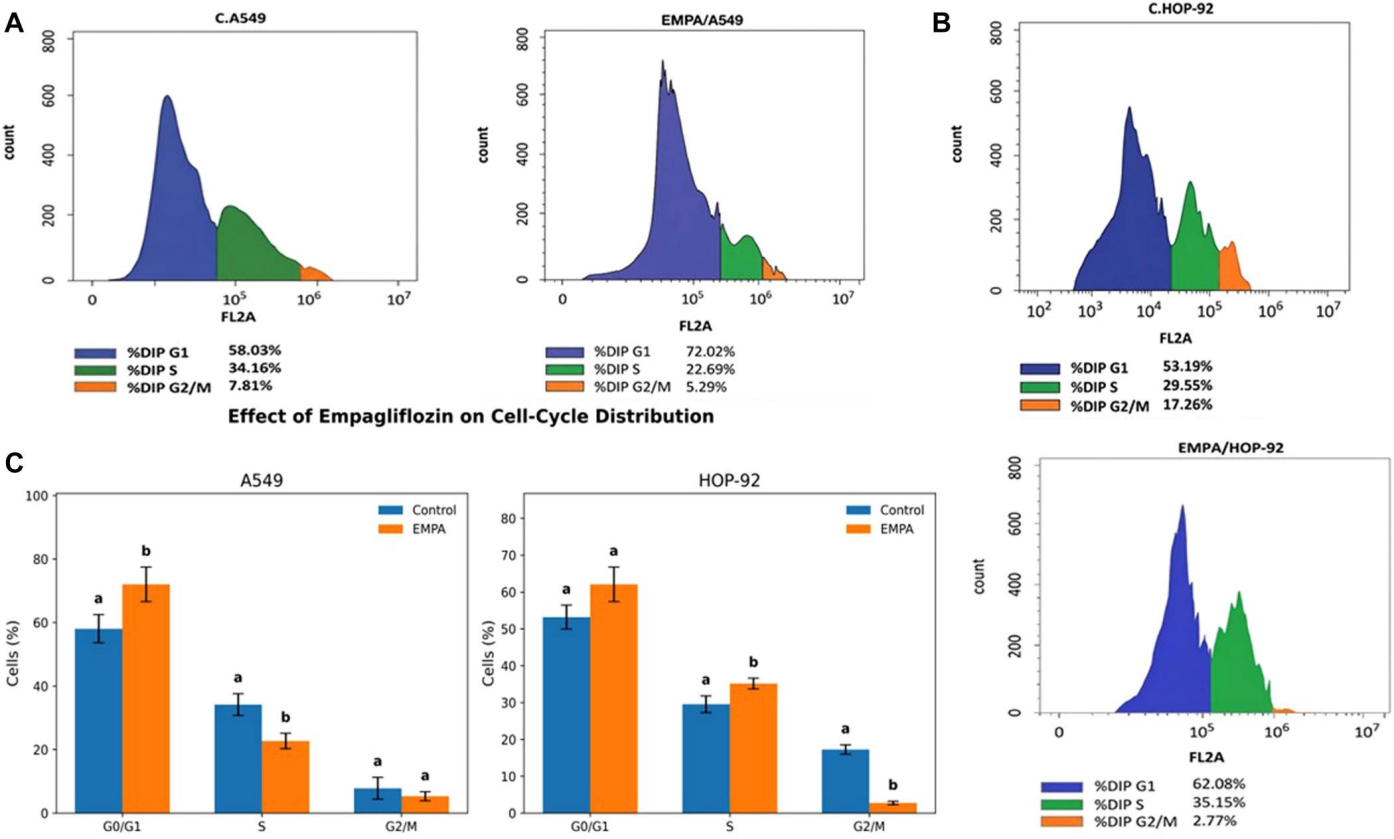
Effect of empagliflozin on cell‐cycle distribution in A549 and HOP‐92 cells. (A, B) Representative flow‐cytometric histograms showing EMPA‐induced cell‐cycle alterations in A549 and HOP‐92 cells, respectively. (C) Quantitative analysis of G0/G1, S, and G2/M populations. Data are mean ± SD (*n* = 3). Different letters indicate significant differences between control and EMPA groups (*p* < 0.05).

A distinct pattern was observed in HOP‐92 cells. EMPA treatment increased the G0/G1 population from **53.19% to 62.08%** and the S‐phase population from **29.55% to 35.15%**, whereas the G2/M population markedly decreased from **17.26% to 2.77%**. The concomitant accumulation of cells in the G0/G1 and S phases, together with substantial depletion of the G2/M population, was reported as **G1/S cell‐cycle arrest** (Table 4, Figure 5).

Collectively, these findings demonstrate that EMPA disrupted cell‐cycle progression in both lung cancer cell lines, although the distributional pattern differed between them. A549 cells showed predominant G0/G1 accumulation with depletion of S‐phase cells, whereas HOP‐92 cells displayed increased representation of both G0/G1 and S phases and pronounced depletion of the G2/M compartment.

#### 3.2.7 Effect of Empagliflozin on Apoptosis and Necrosis

EMPA treatment was also associated with a pronounced increase in cell death in both cell lines. In A549 cells, the total apoptotic/necrotic population increased from **3.28% in control cells to 23.17% following EMPA treatment**. Early apoptosis increased from **0.76% to 3.53%**, while late apoptosis showed a particularly marked increase from **0.29% to 13.39%**. Necrotic cells also increased from **2.23% to 6.25%**. The Annexin V/PI dot plots presented on pages 3–4 visually corroborate the pronounced redistribution of EMPA‐treated A549 cells toward the apoptotic populations (Table 5, Figure 6).

**TABLE 5:** Effect of empagliflozin on apoptosis and necrosis.

Cell line	Treatment	Total cell death (%)	Early apoptosis (%)	Late apoptosis (%)	Necrosis (%)
A549	Control	3.28 ± 0.68^b^	0.76 ± 0.46^a^	0.29 ± 0.43^b^	2.23 ± 0.28^b^
	EMPA	**23.17** ± **2.43^a^**	3.53 ± 1.52^a^	**13.39** ± **2.28^a^**	**6.25** ± **0.79^a^**
HOP‐92	Control	2.95 ± 0.80^b^	0.66 ± 0.52^a^	0.19 ± 0.15^b^	2.10 ± 0.18^b^
	EMPA	**19.43** ± **1.78^a^**	3.52 ± 1.45^a^	**11.30** ± **1.89^a^**	**4.61** ± **0.79^a^**

*Note:* Data are expressed as mean ± SD (*n* = 3). Within each cell line and parameter, values bearing different superscript letters (a, b) are significantly different at *p* < 0.05, whereas values sharing the same letter are not significantly different, as determined using a two‐tailed Welch's *t*‐test.

**FIGURE 6:**
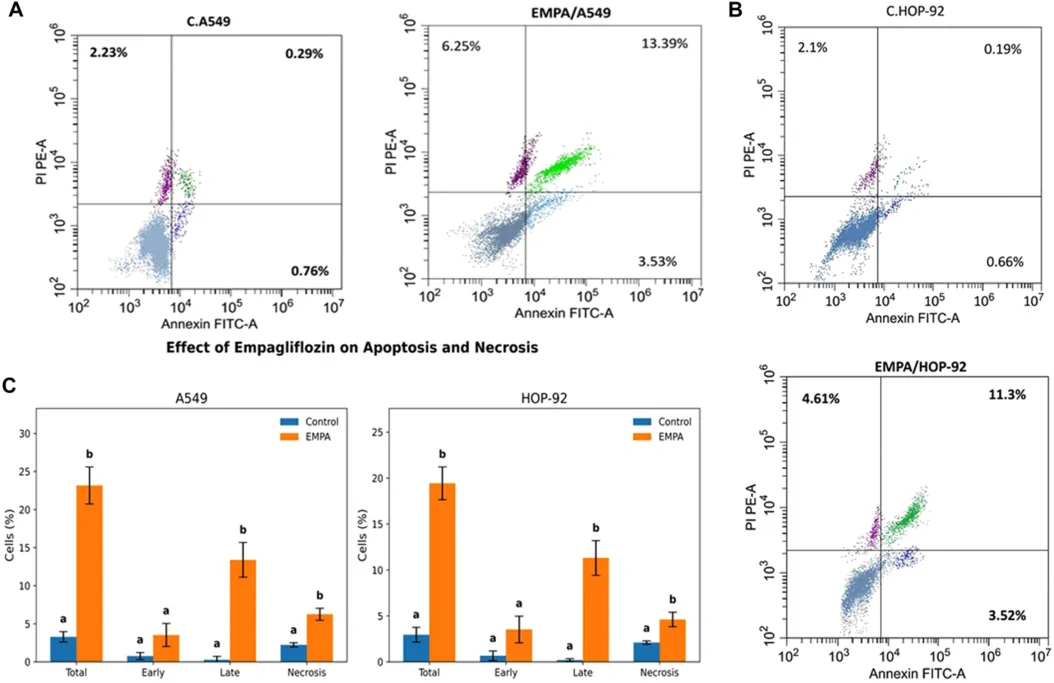
Effect of empagliflozin on apoptosis and necrosis in A549 and HOP‐92 cells. (A, B) Representative Annexin V/PI flow‐cytometry plots of control and EMPA‐treated A549 and HOP‐92 cells, respectively. (C) Quantitative analysis of total apoptosis/cell death, early apoptosis, late apoptosis, and necrosis. Data are mean ± SD (*n* = 3). Different letters indicate significant differences between control and EMPA groups (*p* < 0.05).

A comparable response was observed in HOP‐92 cells. EMPA increased the total apoptotic/necrotic population from **2.95% in control cells to 19.43%**. Early apoptotic cells increased from **0.66% to 3.52%**, whereas late apoptosis increased markedly from **0.19% to 11.30%**. The necrotic population increased from **2.10% to 4.61%**.

Importantly, **late apoptosis constituted the largest cell‐death fraction induced by EMPA in both cell lines**, reaching 13.39% in A549% and 11.30% in HOP‐92 cells. Thus, the flow‐cytometric findings support an EMPA‐associated increase in apoptotic cell death, predominantly characterized by accumulation of cells in the late apoptotic population (Table 5, Figure 6).

**Overall Findings:** Taken together, EMPA treatment produced coordinated alterations in cell‐cycle progression and cell‐death profiles in both A549 and HOP‐92 lung cancer cells. A549 cells exhibited **G1 arrest**, whereas HOP‐92 cells exhibited a **G1/S‐associated arrest pattern**. In parallel, EMPA markedly increased apoptotic cell populations in both models, with late apoptosis representing the predominant cell‐death category.

#### 3.2.8 Effect of Empagliflozin and Hydroxychloroquine on Apoptosis‐ and Autophagy‐Related Gene Expression

RT‐qPCR analysis showed differential regulation of the apoptosis‐related genes **CASP3** and **BAX** and the autophagy‐related gene **SQSTM1/p62** following treatment with empagliflozin (EMPA), hydroxychloroquine (HCQ), and their combination in A549 and HOP‐92 cells (Table 6).

**TABLE 6:** Relative expression of apoptosis‐ and autophagy‐related genes in A549 and HOP‐92 lung cancer cells following treatment with empagliflozin, hydroxychloroquine, and their combination.

Cell line	Treatment	CASP3	BAX	SQSTM1/p62
A549	Control	1.00 ± 0.08^a^	1.00 ± 0.09^a^	1.00 ± 0.08^a^
	EMPA	2.35 ± 0.18ᶜ	2.20 ± 0.17ᶜ	0.82 ± 0.07^a^
	HCQ	1.70 ± 0.14^b^	1.65 ± 0.13^b^	1.55 ± 0.12^b^
	EMPA + HCQ	3.45 ± 0.25^d^	3.10 ± 0.23^d^	2.05 ± 0.16ᶜ
HOP‐92	Control	1.00 ± 0.09^a^	1.00 ± 0.08^a^	1.00 ± 0.09^a^
	EMPA	2.10 ± 0.17ᶜ	1.95 ± 0.16ᶜ	0.95 ± 0.08^a^
	HCQ	1.55 ± 0.13^b^	1.50 ± 0.12^b^	1.65 ± 0.14^b^
	EMPA + HCQ	3.00 ± 0.23^d^	2.70 ± 0.21^d^	2.20 ± 0.18ᶜ

*Note:* Relative mRNA expression of CASP3, BAX, and SQSTM1/p62 was determined by RT‐qPCR and calculated using the 2^−^ΔΔCt method, with the respective untreated control normalized to 1. Data are presented as mean ± SD (*n* = 3). Different superscript letters (a–d) within the same gene and cell line indicate significant differences among treatment groups (*p* < 0.05; one‐way ANOVA followed by Tukey's multiple‐comparison test), whereas values sharing the same letter are not significantly different. BAX, BCL2‐associated X protein; CASP3, caspase‐3; EMPA, empagliflozin; HCQ, hydroxychloroquine; SQSTM1/p62, sequestosome 1.

In **A549 cells**, EMPA increased the relative expression of **CASP3** and **BAX** to 2.35 ± 0.18‐ and 2.20 ± 0.17‐fold, respectively, compared with the control (1.00‐fold). HCQ produced a more moderate increase in CASP3 (1.70 ± 0.14‐fold) and BAX (1.65 ± 0.13‐fold). Notably, combined EMPA + HCQ treatment resulted in the greatest upregulation of both genes, reaching **3.45** ± **0.25‐fold for CASP3 and 3.10** ± **0.23‐fold for BAX**. The treatment groups showed significant differences in both apoptosis‐related genes (*p* < 0.05) (Table 6).

A similar pattern was observed in **HOP‐92 cells**. EMPA increased CASP3 and BAX expression to 2.10 ± 0.17‐ and 1.95 ± 0.16‐fold, respectively, whereas HCQ increased their expression to 1.55 ± 0.13‐ and 1.50 ± 0.12‐fold. The combination treatment produced the highest expression levels, with **CASP3 reaching 3.00** ± **0.23‐fold and BAX reaching 2.70** ± **0.21‐fold**. Thus, the combination produced greater upregulation of apoptosis‐associated genes than either treatment alone (Table 6).

In contrast, **SQSTM1/p62** exhibited a distinct expression pattern. EMPA alone did not significantly alter SQSTM1/p62 expression compared with the respective controls, with values of 0.82 ± 0.07‐fold in A549 and 0.95 ± 0.08‐fold in HOP‐92 cells (*p* > 0.05). HCQ significantly increased SQSTM1/p62 expression to 1.55 ± 0.12‐ and 1.65 ± 0.14‐fold in A549 and HOP‐92 cells, respectively. The greatest upregulation was observed following combined EMPA + HCQ treatment, reaching **2.05** ± **0.16‐fold in A549 and 2.20** ± **0.18‐fold in HOP‐92 cells** (*p* < 0.05).

#### 3.2.9 Empagliflozin Activates Caspase‐3‐Mediated Apoptosis in A549 and HOP‐92 Cells

Flow cytometric analysis demonstrated a marked increase in the percentage of cleaved caspase‐3‐positive cells following treatment with empagliflozin (EMPA) and chloroquine (HCQ). In A549 cells, cleaved caspase‐3 positivity increased from 1.43 ± 0.73% in control cells to 21.61 ± 1.55% following EMPA treatment, while HCQ increased the percentage to 15.82 ± 0.87%. The combined EMPA + HCQ treatment produced the greatest increase, reaching 33.59 ± 1.56% (Table 7, Figure 7).

**TABLE 7:** Effect of empagliflozin, chloroquine, and their combination on cleaved caspase‐3‐positive cells in A549 and HOP‐92 lung cancer cell lines.

Cell line	Control	EMPA	HCQ	EMPA + HCQ
A549	1.43 ± 0.73^a^	21.61 ± 1.55ᶜ	15.82 ± 0.87^b^	33.59 ± 1.56^d^
HOP‐92	2.04 ± 0.56^a^	19.47 ± 1.48ᶜ	12.95 ± 1.29^b^	27.63 ± 1.97^d^

*Note:* Data are presented as mean ± SD (*n* = 3). Different superscript letters (a–d) within the same cell line indicate statistically significant differences among treatment groups (*p* < 0.05), as determined by one‐way ANOVA followed by Tukey's multiple‐comparison test. Groups sharing the same letter are not significantly different. EMPA, empagliflozin; HCQ, chloroquine. Cleaved caspase‐3‐positive cells (%) represent cells expressing the active form of caspase‐3, as determined by flow cytometry.

**FIGURE 7:**
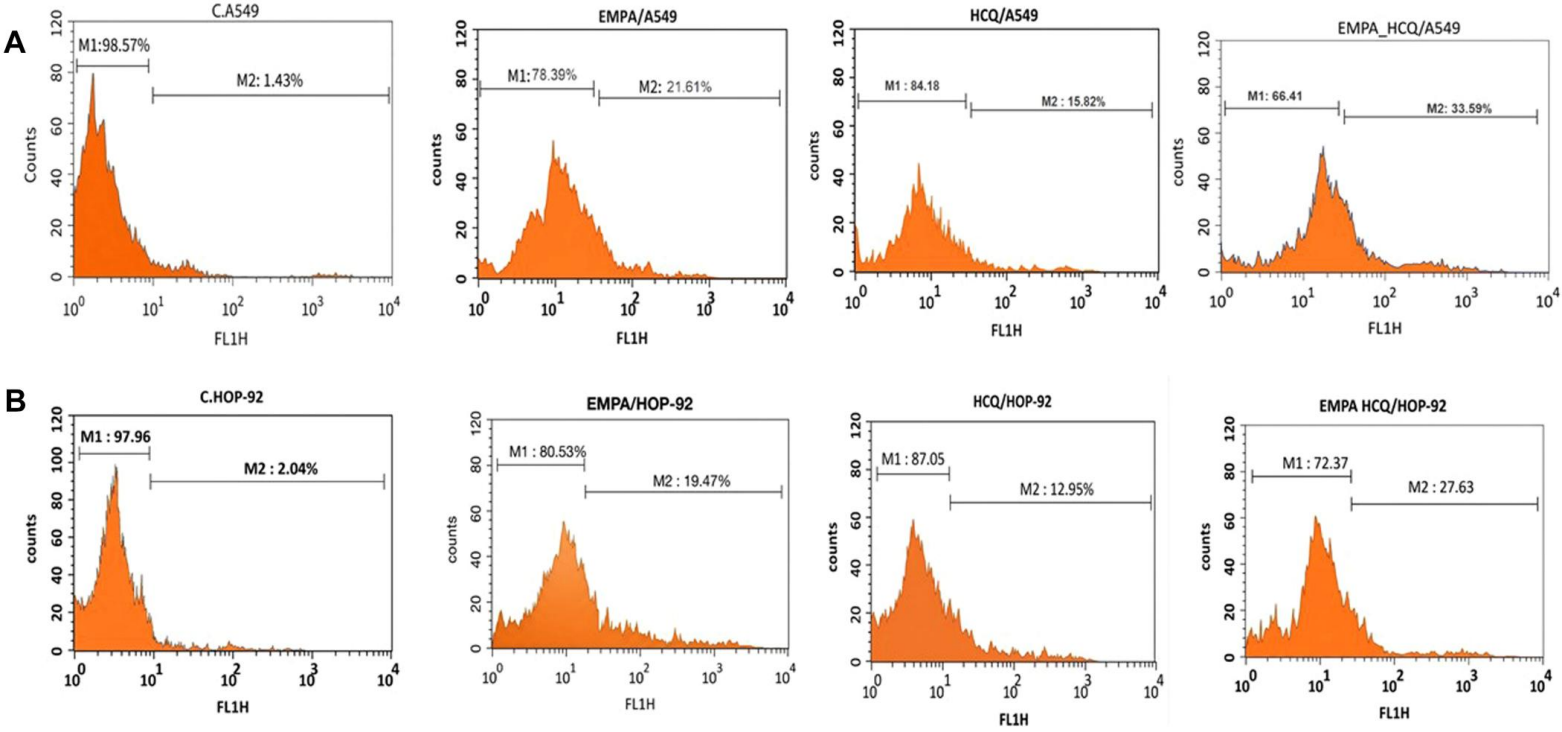
Flow cytometric analysis of cleaved caspase‐3 activation in A549 and HOP‐92 lung cancer cells. Representative histograms showing cleaved (active) caspase‐3‐positive cells in (A) A549 and (B) HOP‐92 cells following treatment with empagliflozin (EMPA), chloroquine (HCQ), or their combination (EMPA + HCQ). M1 represents the cleaved caspase‐3‐negative population, whereas M2 represents cleaved caspase‐3‐positive cells. EMPA and HCQ increased caspase‐3 activation compared with untreated controls, with the highest percentage of cleaved caspase‐3‐positive cells observed following combined EMPA + HCQ treatment in both cell lines.

A comparable response was observed in HOP‐92 cells. Cleaved caspase‐3‐positive cells increased from 2.04 ± 0.56% in the control group to 19.47 ± 1.48% with EMPA and 12.95 ± 1.29% with HCQ. Combination treatment further increased cleaved caspase‐3 positivity to 27.63 ± 1.97%, representing the highest response among the HOP‐92 groups (Table 7, Figure 7).

Collectively, these findings indicate that EMPA promotes activation of the executioner caspase‐3 pathway in both lung cancer cell lines, while concomitant HCQ treatment further enhances caspase‐3 activation, supporting increased apoptotic cell death with the combination.

#### 3.2.10 Effect of Empagliflozin and Hydroxychloroquine on LC3‐II and p62/SQSTM1 Protein Expression

Western blot analysis revealed significant treatment‐dependent alterations in the autophagy‐related proteins **LC3‐II and p62/SQSTM1** in both A549 and HOP‐92 cells. Densitometric values are expressed as **mean ± SD from three replicates (*n*** = **3)**. The uploaded reports show LC3‐II at approximately 14 kDa and p62 at 62 kDa, with β‐actin serving as the loading control (Table 9).

In **A549 cells**, LC3‐II expression increased from **1.538** ± **0.85** in control cells to **5.450** ± **0.97** following EMPA treatment and **3.491** ± **0.65** following HCQ treatment. The highest LC3‐II level was observed with combined EMPA + HCQ treatment (**6.012** ± **1.15**). One‐way ANOVA demonstrated a significant overall treatment effect (*p* = 0.0013). Post‐hoc analysis showed that EMPA significantly increased LC3‐II compared with control, while the combination was significantly higher than both control and HCQ (*p* < 0.05). However, the difference between EMPA and the combination was not significant.

A different pattern was observed for **p62/SQSTM1 in A549 cells**. EMPA slightly reduced p62 from **2.079** ± **0.77** in the control to **1.823** ± **0.64**, whereas HCQ increased its level to **4.087** ± **1.02**. The highest p62 expression was detected following combined EMPA + HCQ treatment (**5.495** ± **1.26**), with a significant overall treatment effect (*p* = 0.0044). The combination was significantly higher than both the control and EMPA groups (*p* < 0.05) (Table 8, Figure 8).

**TABLE 8:** Effect of empagliflozin and hydroxychloroquine on LC3‐II and p62/SQSTM1 protein expression in A549 and HOP‐92 lung cancer cells.

Cell line	Treatment	LC3‐II	p62/SQSTM1
A549	Control	1.538 ± 0.85^a^	2.079 ± 0.77^ab^
	EMPA	5.450 ± 0.97ᶜ	1.823 ± 0.64^a^
	HCQ	3.491 ± 0.65^ab^	4.087 ± 1.02^b^ᶜ
	EMPA + HCQ	**6.012** ± **1.15ᶜ**	**5.495** ± **1.26ᶜ**
HOP‐92	Control	1.332 ± 0.99^a^	1.545 ± 0.85^a^
	EMPA	4.639 ± 1.85^ab^	1.585 ± 0.97^a^
	HCQ	3.247 ± 1.24^ab^	4.077 ± 1.34^ab^
	EMPA + HCQ	**5.233** ± **1.25^b^**	**5.602** ± **1.19^b^**

*Note:* Data are presented as **mean ± SD (*n*** = **3)**. Different superscript letters within the same protein and cell line indicate statistically significant differences among treatment groups (*p* < 0.05), based on one‐way ANOVA followed by Tukey's multiple‐comparison test; groups sharing at least one letter are not significantly different. **EMPA**, empagliflozin; **HCQ**, hydroxychloroquine; **LC3‐II**, microtubule‐associated protein 1 light chain 3‐II; **p62/SQSTM1**, sequestosome 1. Protein expression was assessed by Western blotting, with β‐actin used as the loading control.

**FIGURE 8:**
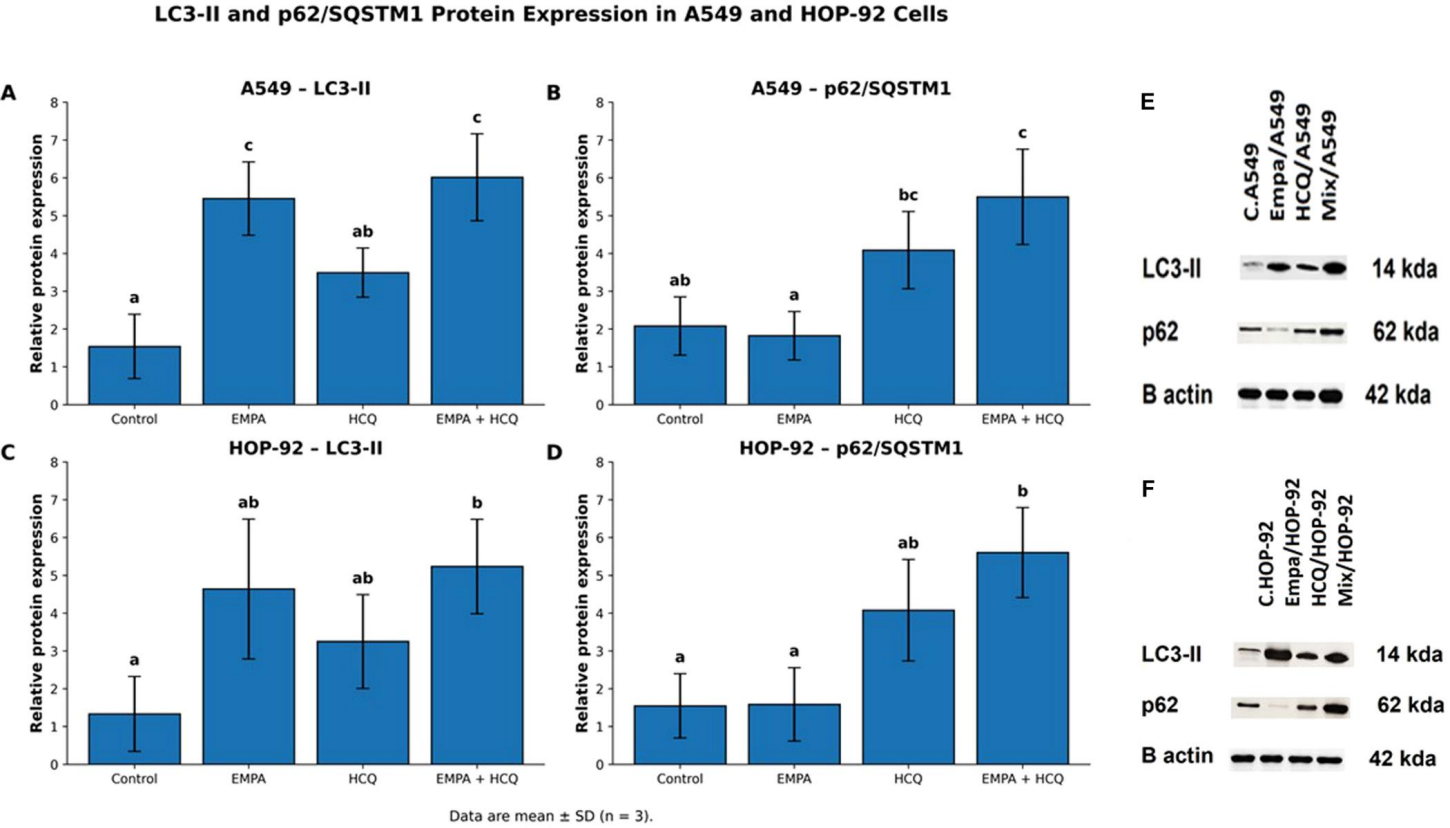
Effect of empagliflozin and hydroxychloroquine on LC3‐II and p62/SQSTM1 protein expression in A549 and HOP‐92 lung cancer cells. Densitometric analysis of LC3‐II (A, C) and p62/SQSTM1 (B, D) protein expression in A549 (A, B) and HOP‐92 (C, D) cells following treatment with empagliflozin (EMPA), hydroxychloroquine (HCQ), or their combination (EMPA + HCQ). (E, F) Representative Western blot images of LC3‐II (~14 kDa), p62/SQSTM1 (~62 kDa), and β‐actin (~42 kDa) in A549 and HOP‐92 cells, respectively. Protein levels were normalized to β‐actin. Data are presented as mean ± SD (*n* = 3). Different superscript letters indicate significant differences among treatment groups within each panel (*p* < 0.05; one‐way ANOVA followed by Tukey's multiple‐comparison test), whereas groups sharing at least one letter are not significantly different.

In **HOP‐92 cells**, LC3‐II increased from **1.332** ± **0.99** in the control group to **4.639** ± **1.85** following EMPA and **3.247** ± **1.24** following HCQ treatment. Again, the highest level was detected in the EMPA + HCQ group (**5.233** ± **1.25**). The overall treatment effect was significant (*p* = 0.0337), although the Tukey post‐hoc analysis identified a significant difference specifically between the control and combination groups (*p* < 0.05) (Table 8, Figure 8).

For **p62/SQSTM1 in HOP‐92 cells**, EMPA alone produced little change (**1.585** ± **0.97**) relative to control (**1.545** ± **0.85**). In contrast, HCQ markedly increased p62 to **4.077** ± **1.34**, while the combination produced the highest level (**5.602** ± **1.19**). The overall treatment effect was significant (*p* = 0.0048), and p62 expression in the combination group was significantly higher than in both control and EMPA‐treated cells (*p* < 0.05) (Table 8, Figure 8).

Collectively, EMPA was associated with **LC3‐II accumulation without corresponding p62 accumulation**, particularly in A549 cells, whereas HCQ was associated with accumulation of both LC3‐II and p62. The combination produced the **highest numerical levels of both markers in both cell lines**. This pattern is consistent with EMPA‐associated enhancement of autophagic activity and HCQ‐mediated inhibition of downstream lysosomal degradation. Importantly, the study protocol notes that LC3‐II and p62 should be interpreted together because LC3‐II accumulation alone cannot distinguish increased autophagosome formation from impaired autophagic degradation.

## 4 Discussion

The present study investigated the anticancer potential of empagliflozin (EMPA) against lung cancer through an integrated approach combining network pharmacology, molecular docking, and experimental validation in A549 and HOP‐92 cells. The findings demonstrate that EMPA exerted concentration‐dependent cytotoxicity, disrupted cell‐cycle progression, and promoted apoptotic cell death. These effects were accompanied by increased BAX and CASP3 expression, enhanced cleaved caspase‐3 activation, and modulation of the autophagy‐associated proteins LC3‐II and p62/SQSTM1. Importantly, hydroxychloroquine (HCQ) markedly enhanced EMPA‐mediated cytotoxicity and apoptotic signaling while promoting accumulation of LC3‐II and p62. Collectively, these findings suggest that the anticancer activity of EMPA involves coordinated regulation of proliferation, apoptosis, and autophagy.

Network pharmacology identified 239 common targets, of which 46 were retained after applying an association‐score cutoff of 0.30 between EMPA‐associated and lung cancer‐related genes, including highly connected proteins involved in tumor survival and signaling, such as EGFR, NFKB1, MAPK1, MAPK3, and MTOR, together with apoptosis‐ and autophagy‐associated targets including **BAX, CASP3, SQSTM1,** and MAP1LC3B. These findings are biologically relevant because dysregulation of MAPK and mTOR signaling, apoptosis, metabolism, and autophagy contributes substantially to NSCLC development and therapeutic resistance [1, 3]. Recent evidence further suggests that SGLT2 inhibitors may influence cancer‐cell metabolism, proliferation, survival, and intracellular signaling beyond their established glucose‐lowering actions [33].

Molecular docking indicated that EMPA can be geometrically accommodated at surface sites of ANXA8 (−7.6 kcal/mol) and the p62/SQSTM1 ZZ domain (−7.0 kcal/mol) and within the ligand‐binding pocket of PPARγ (−7.5 kcal/mol), values that were reproduced by independent re‐docking with a protocol validated against a crystallographic pose (RMSD 1.30 ± 0.02 Å; Table 3). p62/SQSTM1 is of mechanistic interest because it acts both as a selective autophagy receptor and as a signaling adaptor linking autophagy with stress responses, survival and apoptosis (Moscat & Díaz‐Meco, 2009), and the PPARγ result is consistent with the changes in PPARG expression measured here and with the reported metabolic and transcriptional effects of SGLT2 inhibitors [33]. These calculations do not, however, establish direct target engagement: in the same validated EGFR site, EMPA scored more favorably than erlotinib while having no measured EGFR activity, so the scores are best read as plausibility estimates requiring biophysical confirmation.

Experimentally, EMPA produced concentration‐dependent cytotoxicity in both lung cancer cell lines, with IC_50_ values of 52.905 ± 1.85 µg/mL in A549 and 81.629 ± 2.50 µg/mL in HOP‐92 cells, indicating greater sensitivity of A549 cells. These findings are consistent with emerging evidence supporting anticancer effects of SGLT2 inhibitors. EMPA and related agents have been reported to interfere with cancer‐cell proliferation and metabolic signaling in different experimental tumor models [7, 8, 33]. In particular, recent evidence indicates that SGLT2 inhibitors can influence AMPK/Akt/mTOR‐related signaling, which is relevant to the present network analysis identifying MTOR among the central targets [8]. The difference in sensitivity between A549 and HOP‐92 cells may reflect tumor‐cell heterogeneity in metabolic dependence, transporter expression, survival signaling, or basal autophagic activity.

A major finding of the present study was the substantial enhancement of EMPA cytotoxicity by HCQ. Combined treatment reduced the IC_50_ to 11.286 ± 0.36 µg/mL in A549 and 28.919 ± 1.08 µg/mL in HOP‐92 cells. Chou–Talalay analysis yielded CI_50_ values of 0.406 and 0.618, respectively, indicating synergism at the 50% effect level [25]. However, Bliss analysis showed concentration‐dependent interactions, with stronger enhancement at low‐to‐intermediate concentrations and weaker or antagonistic interactions at higher concentrations. Thus, the interaction should be interpreted as dose dependent rather than uniformly synergistic. This distinction is important because conclusions regarding drug synergy can vary according to concentration and the mathematical reference model employed [25, 34].

The enhanced activity of HCQ may be related to its ability to interfere with lysosomal autophagic degradation. Autophagy can provide established cancer cells with metabolic substrates and facilitate adaptation to therapeutic stress, whereas pharmacological inhibition of autophagy may increase sensitivity to anticancer agents (Levy et al., 2017; [35]). Indeed, recent evidence indicates that autophagy inhibition can augment responses to chemotherapy, targeted therapy, and other anticancer approaches, although clinical results with first‐generation lysosomal inhibitors such as HCQ have been variable [35].

In addition to reducing cell viability, EMPA disrupted cell‐cycle progression. In A549 cells, EMPA increased the G0/G1 population from 58.03% to 72.02%, accompanied by a reduction in S‐phase cells, supporting predominant G1 arrest. In HOP‐92 cells, G0/G1 increased from 53.19% to 62.08%, S phase increased from 29.55% to 35.15%, and G2/M markedly decreased from 17.26% to 2.77%, indicating disruption of progression across the G1/S region. These findings demonstrate that EMPA suppresses proliferation partly through interference with cell‐cycle progression, although the precise response differs between the two cell lines. Such variability is consistent with the molecular and metabolic heterogeneity characteristic of NSCLC [1].

Annexin V/PI analysis further demonstrated that EMPA‐induced cytotoxicity was accompanied predominantly by apoptotic cell death. Total cell death increased from 3.28% to 23.17% in A549 and from 2.95% to 19.43% in HOP‐92 cells. Late apoptosis represented the largest treatment‐associated fraction, reaching 13.39% and 11.30%, respectively. Although necrosis also increased, its magnitude was considerably lower, supporting apoptosis as an important mechanism of EMPA‐induced cell death. This interpretation was further supported by the molecular analyses.

EMPA significantly increased BAX and CASP3 expression in both cell lines. In A549 cells, BAX and CASP3 increased to 2.20‐ and 2.35‐fold, respectively, whereas in HOP‐92 cells they reached 1.95‐ and 2.10‐fold. BAX is an important regulator of mitochondrial outer‐membrane permeabilization, while caspase‐3 functions as a major executioner of apoptosis (Elmore 2007; Wong 2011). Notably, combined EMPA + HCQ treatment produced the greatest expression of both genes, suggesting enhancement of pro‐apoptotic signaling when lysosomal/autophagic function was inhibited.

This conclusion was strengthened by the cleaved caspase‐3 results. EMPA increased cleaved caspase‐3‐positive cells from 1.43% to 21.61% in A549 and from 2.04% to 19.47% in HOP‐92 cells. The combination with HCQ further increased these values to 33.59% and 27.63%, respectively. Because caspase‐3 becomes functionally active following proteolytic cleavage, these findings provide more direct evidence of apoptotic execution than CASP3 transcription alone (Elmore 2007). The agreement among Annexin V/PI staining, BAX/CASP3 upregulation, and cleaved caspase‐3 activation therefore provides convergent evidence that EMPA promotes caspase‐associated apoptosis in both lung cancer models.

The autophagy findings provide additional mechanistic insight. EMPA markedly increased LC3‐II in both cell lines, whereas p62 decreased slightly in A549 cells and remained relatively unchanged in HOP‐92 cells. LC3‐II accumulation indicates increased abundance of autophagosomes but cannot, when considered alone, distinguish increased autophagosome formation from impaired degradation. Accordingly, LC3‐II should be interpreted together with autophagic substrates such as p62 and preferably in the presence and absence of lysosomal inhibition [5, 32]. The increase in LC3‐II without corresponding p62 accumulation following EMPA treatment is therefore compatible with increased autophagy‐associated activity rather than simple suppression of autophagy.

In contrast, HCQ increased p62 and LC3‐II, while combined EMPA + HCQ treatment produced the highest numerical accumulation of both proteins. Simultaneous accumulation of LC3‐II and p62 is consistent with impaired downstream autophagic degradation because HCQ interferes with lysosomal function and prevents efficient clearance of autophagic cargo [5, 35]. The stronger accumulation observed during combined treatment therefore supports the interpretation that EMPA stimulates an autophagy‐associated response that is subsequently interrupted at the lysosomal degradation stage by HCQ.

The SQSTM1 transcriptional results further emphasize the complexity of p62 regulation. EMPA alone did not significantly alter SQSTM1 mRNA, despite changes in LC3‐II and p62 protein, whereas HCQ and particularly EMPA + HCQ increased SQSTM1 transcription. Because p62 abundance depends on both transcriptional regulation and degradation through autophagy, SQSTM1 mRNA and p62 protein are not necessarily expected to change in parallel (Moscat and Díaz‐Meco 2009; [5]). Thus, evaluation of both transcriptional and protein‐level changes provides a more complete interpretation of the autophagic response.

Integration of the apoptosis and autophagy results suggests a potentially important interaction between these processes. Autophagy and apoptosis form interconnected stress‐response pathways, and autophagy can either protect cancer cells from apoptosis or contribute to cell death depending on the intensity and duration of stress and the molecular context [36]. This dual role is particularly relevant in lung cancer, where treatment‐induced autophagy may act as an adaptive mechanism that supports tumor‐cell survival, while inhibition of protective autophagy can increase apoptotic susceptibility [36].

The present findings are consistent with a model in which EMPA induces cellular stress that simultaneously activates apoptotic signaling and an autophagy‐associated adaptive response. When downstream autophagic degradation was disrupted by HCQ, EMPA‐mediated cytotoxicity increased substantially, while BAX, CASP3, and cleaved caspase‐3 responses were further enhanced. This pattern suggests that autophagy may, at least partly, provide an adaptive survival mechanism during EMPA exposure and that interference with this pathway shifts the cellular response toward apoptotic execution. This interpretation is consistent with recent evidence supporting autophagy inhibition as a strategy for sensitizing established tumors to anticancer treatments [35, 36]. However, because autophagy can exert cytoprotective or cytotoxic functions depending on cellular context, a direct causal role should not be concluded from the present experiments alone [36].

Overall, the agreement between the computational predictions and experimental findings supports a multitarget mechanism for EMPA in lung cancer. Network pharmacology identified apoptosis‐, autophagy‐, and survival‐related targets, while experimental analyses demonstrated inhibition of proliferation, cell‐cycle disruption, activation of BAX/caspase‐3‐associated apoptosis, and modulation of LC3‐II/p62 signaling. Recent literature similarly emphasizes that SGLT2 inhibitors may affect tumor metabolism and signaling through mechanisms extending beyond glucose transport inhibition [33]. The enhanced activity observed with HCQ further suggests that autophagic/lysosomal adaptation may influence the response of lung cancer cells to EMPA.

Collectively, the present findings demonstrate that EMPA exerts antiproliferative and pro‐apoptotic effects in A549 and HOP‐92 lung cancer cells while simultaneously inducing an autophagy‐associated response. Pharmacological interference with lysosomal autophagic degradation by HCQ markedly enhanced EMPA‐mediated cytotoxicity and apoptotic signaling. These findings suggest functional crosstalk between autophagy and apoptosis during EMPA treatment and provide a rationale for further investigation of EMPA combined with autophagy‐targeting strategies in lung cancer.

## 5 Conclusion

The present study demonstrates that empagliflozin (EMPA) exerts antiproliferative and pro‐apoptotic effects in A549 and HOP‐92 lung cancer cells, supported by concentration‐dependent reductions in cell viability, disruption of cell‐cycle progression, increased apoptotic cell populations, upregulation of BAX and CASP3, and enhanced activation of cleaved caspase‐3. The integrated network pharmacology and molecular docking analyses further suggest that EMPA may act through multiple interconnected pathways involved in tumor survival, apoptosis, metabolism, and autophagy.

Importantly, EMPA markedly increased LC3‐II without corresponding p62/SQSTM1 accumulation, a pattern consistent with enhanced autophagy‐associated activity. In contrast, hydroxychloroquine (HCQ), particularly when combined with EMPA, promoted accumulation of both LC3‐II and p62, consistent with inhibition of downstream lysosomal autophagic degradation. The EMPA + HCQ combination also markedly reduced the concentrations required for cytotoxicity and enhanced BAX/CASP3 expression and cleaved caspase‐3 activation compared with either treatment alone. These findings suggest that autophagy may function, at least partly, as an adaptive response to EMPA‐induced cellular stress, while its pharmacological disruption enhances apoptotic cell death.

Overall, the study reveals functional crosstalk between autophagy and apoptosis during EMPA treatment and provides a mechanistic rationale for further investigation of EMPA in combination with autophagy‐targeting approaches in lung cancer. Nevertheless, validation using additional lung cancer cell models and *in vivo* studies is required before the therapeutic relevance of this combination.

## Author Contributions

**Ola A El‐Feky:** conceptualization, investigation, methodology, software, supervision, formal analysis, visualization. **Sara M Hamouda:** investigation, writing – original draft, visualization, methodology, formal analysis, data curation. **Manar Mohammed El Tabaa:** conceptualization, investigation, writing – original draft, methodology, validation, visualization, software, data curation. **Sally E Abu‐Risha:** supervision, software, writing – review and editing, writing – original draft. **Ahmed B M Mehany:** methodology, validation, visualization, writing – original draft. **Othman F Abdelzaher:** investigation, writing – original draft, validation, methodology, visualization. **Samar A El‐Adawy:** investigation, methodology, validation, visualization, formal analysis, writing – original draft, writing – review and editing.

## Funding

The authors have nothing to report.

## Ethics Statement

All in‐vitro analysis procedures were performed under aseptic conditions according to standard policies of GENE TECH Medical, Genetic & Scientific Research Center, El‐Mansoura, Egypt. Cell line cultures and treatments were performed under ethical considerations of Ethics Research Committee, Faculty of Pharmacy, Tanta University (TP/RE/10/25 p‐006).

## Supporting information


**Figure S1:** Open Targets association scores of the top‐10 hub targets and the experimentally validated targets. Overall association scores for lung neoplasm (MONDO_0021117) are shown for the ten PPI hub targets identified by CytoNCA (light filled circles) and the seven experimentally validated targets (dark filled circles). Open circles denote targets whose association rests on text‐mining evidence alone. The dashed line marks the 0.30 cutoff, which is exceeded by 9 of the 17 targets. Score and rank among the 16,501 associated targets are annotated for selected genes: *EGFR* 0.90 (rank 1), *PPARGC1A* 0.11 (rank 1,468), *SQSTM1* 0.10 (rank 2,185) and *ANXA8* 0.04 (rank 6,187).

Supporting File 1

Supporting File 2

Supporting File 3

Supporting File 4

Supporting File 5

Supporting File 6

Supporting File 7

Supporting File 8

Supporting File 9

Supporting File 10

Supporting File 11

Supporting File 12

## Data Availability

The target proteins annexin A8 (UniProt P13928; PDB 1W3W), the ZZ‐type zinc‐finger domain of p62/SQSTM1 (UniProt Q13501; PDB 5YP7) and the PPARγ ligand‐binding domain in complex with a PGC‐1α coactivator peptide (UniProt P37231 and Q9UBK2; PDB 8BF1) were selected, and their structures were obtained from the RCSB Protein Data Bank (https://www.rcsb.org/); the EGFR kinase domain in complex with erlotinib (PDB 1M17) was used for protocol validation. These are well‐characterized proteins with publicly available sequences; no new protein sequence data were generated in this study. Empagliflozin was obtained from PubChem (CID 11949646). Lung‐cancer target associations were retrieved from the Open Targets Platform (MONDO_0021117) through its public GraphQL API, and reference‐ligand potency data from ChEMBL. All docking parameters, output modes and docked pose coordinates are provided as Tables S5 and S6 and as supplementary structure files; the target‐filtering script is provided as Supporting Information S1: File 1.
